# Epoxinnamide: An Epoxy Cinnamoyl-Containing Nonribosomal Peptide from an Intertidal Mudflat-Derived *Streptomyces* sp.

**DOI:** 10.3390/md20070455

**Published:** 2022-07-12

**Authors:** Sangwook Kang, Jaeho Han, Sung Chul Jang, Joon Soo An, Ilnam Kang, Yun Kwon, Sang-Jip Nam, Sang Hee Shim, Jang-Cheon Cho, Sang Kook Lee, Dong-Chan Oh

**Affiliations:** 1 Natural Products Research Institute, College of Pharmacy, Seoul National University, Seoul 08826, Korea; ksw1657@snu.ac.kr (S.K.); gh03292@snu.ac.kr (J.H.); tobok95@snu.ac.kr (S.C.J.); ahnjunsoo@snu.ac.kr (J.S.A.); sanghee_shim@snu.ac.kr (S.H.S.); sklee61@snu.ac.kr (S.K.L.); 2 Department of Biological Sciences, Inha University, Incheon 22212, Korea; ikang@inha.ac.kr (I.K.); chojc@inha.ac.kr (J.-C.C.); 3 Research Institute of Pharmaceutical Sciences, College of Pharmacy, Kyungpook National University, Daegu 41566, Korea; yunkwon@knu.ac.kr; 4 Department of Chemistry and Nanoscience, Ewha Womans University, Seoul 03760, Korea; sjnam@ewha.ac.kr

**Keywords:** cinnamoyl-containing nonribosomal peptide, *Streptomyces*, biosynthetic gene cluster, bifunctional thioesterase, quinone reductase, angiogenesis

## Abstract

Cinnamoyl-containing nonribosomal peptides (CCNPs) form a unique family of actinobacterial secondary metabolites and display various biological activities. A new CCNP named epoxinnamide (**1**) was discovered from intertidal mudflat-derived *Streptomyces* sp. OID44. The structure of **1** was determined by the analysis of one-dimensional (1D) and two-dimensional (2D) nuclear magnetic resonance (NMR) data along with a mass spectrum. The absolute configuration of **1** was assigned by the combination of advanced Marfey’s method, ^3^*J*_HH_ and rotating-frame overhauser effect spectroscopy (ROESY) analysis, DP4 calculation, and genomic analysis. The putative biosynthetic pathway of epoxinnamide (**1**) was identified through the whole-genome sequencing of *Streptomyces* sp. OID44. In particular, the thioesterase domain in the nonribosomal peptide synthetase (NRPS) biosynthetic gene cluster was proposed as a bifunctional enzyme, which catalyzes both epimerization and macrocyclization. Epoxinnamide (**1**) induced quinone reductase (QR) activity in murine Hepa-1c1c7 cells by 1.6-fold at 5 μM. It also exhibited effective antiangiogenesis activity in human umbilical vein endothelial cells (IC_50_ = 13.4 μM).

## 1. Introduction

Numerous natural products with interesting biological activities are biosynthesized by nonribosomal peptide synthases (NRPSs) and polyketide synthases (PKSs). The assembly of NRPSs/PKSs has been logically understood as iterative elongation of monomer units orchestrated by key domains (C: condensation, A: adenylation, PCP: peptidyl carrier protein for NRPSs/KS: ketosynthase, AT: acyltransferase, ACP: acyl carrier protein for PKSs), as illustrated in Figure S1 [1].

Among various nonribosomal peptides, cinnamoyl-containing nonribosomal peptides (CCNPs) form a small family of bacterial secondary metabolites from actinomycetes (Figure S2). Natural products in this family are structurally and biologically interesting because they have unique cinnamoyl moieties as acyl chains and show various biological activities [2]. The family includes WS9326s (tachykinin antagonist and its derivatives from *Streptomyces violaceusniger* No. 9326, *Streptomyces asterosporus* DSM 41452, and *Streptomyces* sp. 9078) [3,4,5], pepticinnamin E (a farnesyl-protein transferase inhibitor from *Streptomyces* sp. OH-4652) [6], skyllamycins (platelet-derived growth factor signaling pathway inhibitor and its derivatives from *Streptomyces* sp. KY11784, *Streptomyces* sp. Acta 2897, *Streptomyces* sp. strain 1675, and *Streptomyces anulatus*) [7,8,9,10], eudistamides (antibiotics from *Streptomyces* sp. WMMB 705) [11], NC-1 (an antitubercular agent from *Streptomyces* sp. FXJ1.172) [12], atratumycin (an antitubercular agent from *Streptomyces atratus* SCSIO ZH16) [13], atrovimycin (an antitubercular and antifungal agent from *Streptomyces atrovirens* LQ13) [14], kitacinnamycins (stimulators of interferon genes activator from *Kitasatospora* sp. CGMCC 16924) [2], cinnapeptin (an antibiotic from *Streptomyces ghanaensis*) [15], and nyuzenamides A and B (antifungal and cytotoxic agents from *Streptomyces* sp. N11-34) [16]. We reported mohangamides A and B (*Candida albicans* isocitrate lyase inhibitors) [17] and WS9326H (an antiangiogenic peptide) [18] from *Streptomyces* sp. SNM55, coprisamides (quinone reductase inducers and antitubercular agents) from *Streptomyces* sp. SNU533 and *Micromonospora* sp. UTJ3 [19,20], and nyuzenamide C (an antiangiogenesis agent and quinone reductase inducer) from *Streptomyces* sp. DM14 [21].

**Figure 1:**
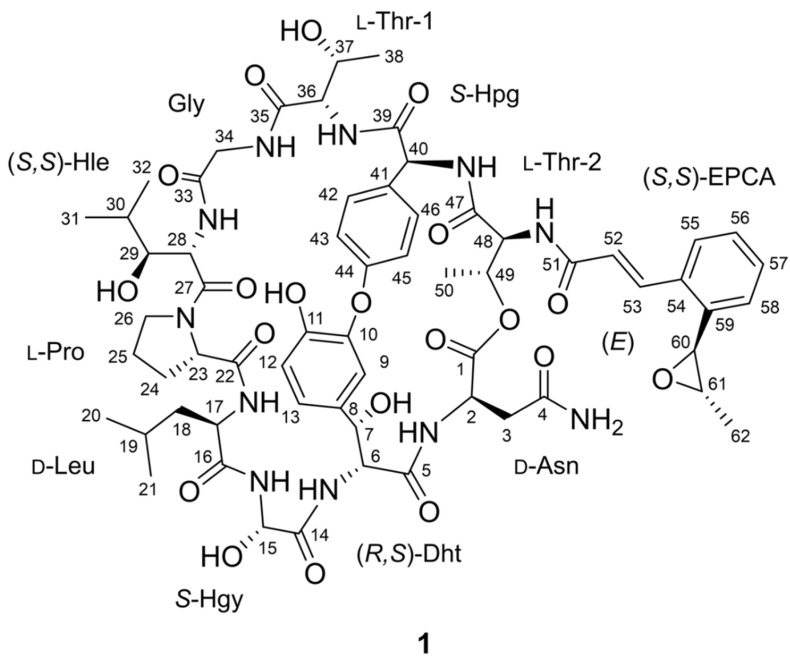
The structure of epoxinnamide (**1**). Dht = 3,*β*-dihydroxytyrosine, Hgy = *α*-hydroxyglycine, Hle = *β*-hydroxyleucine, Hpg = hydroxyphenylglycine, EPCA = *O*-1,2-epoxypropyl cinnamic acid.

Marine ecosystems are the largest aquatic environments on Earth and provide diverse habitats for various marine organisms. As over 1400 new marine natural products were discovered in 2020 [22], marine ecosystems are considered rich sources of new bioactive compounds, including marine actinomycete-derived CCNPs (skyllamycin B, eudistamides, atratumycin, WS9326H and mohangamides). In this study, we performed chemical screening of secondary metabolites of 192 actinomycete strains, which were isolated from the intertidal mudflat in Oido, Siheung city on the west coast of Korea, based on the analysis of liquid chromatography/mass spectrometry (LC/MS) profiles. We found a bacterial strain, *Streptomyces* sp. OID44, which produces a major compound with the ultraviolet (UV) spectrum similar to those of previously reported CCNPs but with a distinct pseudomolecular ion [M + H]^+^ at *m*/*z* 1298.5611. Subsequent scaling up of the culture, chromatographic purification, and spectroscopic analysis of the major compound allowed us to characterize a new CCNP, epoxinnamide (**1**) (Figure 1). Here, we report the structure, putative biosynthetic pathway, and biological activities of **1**.

## 2. Results

### 2.1. Structure Elucidation and Putative Biosynthetic Pathway of Epoxinnamide *(**1**)*

Epoxinnamide (**1**), a white amorphous powder, displayed a UV absorption peak at a 280 nm wavelength. Its formula was assigned as C_62_H_79_N_11_O_20_ (29 degrees of unsaturation) by high-resolution electrospray ionization mass spectrometry (HRESIMS) (Figure S3) along with the ^1^H and ^13^C nuclear magnetic resonance (NMR) spectroscopic data (Figures S4 and S5). The ^1^H and heteronuclear single quantum coherence (HSQC) NMR data (Figure S6) indicated that **1** has 16 exchangeable protons (*δ*_H_ 9.40, 9.07, 8.96, 8.44, 8.23, 8.16, 8.14, 7.69, 7.68, 7.55, 7.43, 7.17, 7.03, 6.15, 5.09, and 4.52), 11 aromatic protons (*δ*_H_ 7.42, 7.37, 7.33, 7.18, 7.09, 7.08, 7.07, 7.00, 6.87, 6.42, and 6.27), 2 *trans*-coupling olefinic protons (*δ*_H_ 8.11 and 6.94; *J* = 15.5 Hz), and 19 protons bound to heteroatom-bearing carbons (*δ*_H_ 5.79, 5.19, 5.03, 4.83, 4.69, 4.64, 4.59, 4.44, 4.40, 4.37, 4.18, 4.07, 3.99, 3.72, 3.52, 3.49, 3.39, 3.22, and 2.97). In addition, 2 aliphatic methine protons (*δ*_H_ 1.74 and 1.46), 8 aliphatic methylene protons (*δ*_H_ 2.65–1.31), and 21 methyl protons (*δ*_H_ 1.37–0.72) were observed (Table 1).

The ^13^C NMR data (Figure S5) showed that **1** has 12 carbonyl carbons (*δ*_C_ 173.3, 171.8, 171.5, 170.9, 170.64, 170.63, 170.1, 170.0, 169.6, 169.2, 169.1, and 168.4), 3 heteroatom-bound aromatic carbons (*δ*_C_ 159.6, 147.4, and 147.3), an additional 15 aromatic carbons (*δ*_C_ 136.74, 134.1, 132.3, 131.6, 131.3, 129.6, 128.5, 127.8, 126.9, 124.4, 123.6, 122.3, 120.2, 120.1, and 116.1), two olefinic carbons (*δ*_C_ 136.69 and 124.6), and 17 N/O-bound sp^3^ carbons (*δ*_C_ 74.4, 71.3, 69.5, 68.7, 65.8, 63.8, 61.0, 60.2, 60.1, 58.9, 57.7, 54.8, 52.5, 50.3, 49.0, 47.3, and 42.60). Besides these resonances, two methine carbons (*δ*_C_ 27.9, and 24.1), four methylene carbons (*δ*_C_ 42.58–24.7), and seven methyl carbons (*δ*_C_ 23.2–14.4) were identified as aliphatic signals. All the 1-bond C-H correlations were assigned by ^1^H, ^13^C, and HSQC NMR spectroscopic analysis (Table 1).

As **1** showed a UV spectrum very similar to nyuzenamide C, we compared the ^1^H and ^13^C NMR data of the two compounds (Figure S7, Table S1) [21]. Our spin system analysis based on correlation spectroscopy (COSY) and total correlation spectroscopy (TOCSY) data (Figures S8 and S9) revealed that **1** shared eight identical amino acid units with nyuzenamide C, asparagine (Asn), 3,*β*-dihydroxytyrosine (Dht), *α*-hydroxyglycine (Hgy), leucine (Leu), proline (Pro), glycine (Gly), hydroxyphenylglycine (Hpg) and threonine (Thr-2) (Figure S7). The carbonyl groups of these amino acid residues were assigned by analyzing the heteronuclear multiple bond correlation (HMBC) data (Figure S10). However, *β*-hydroxyphenylalanine and valine units in nyuzenamide C were not identified in the NMR data of **1**, thus requiring further comprehensive analysis.

**Figure 2:**
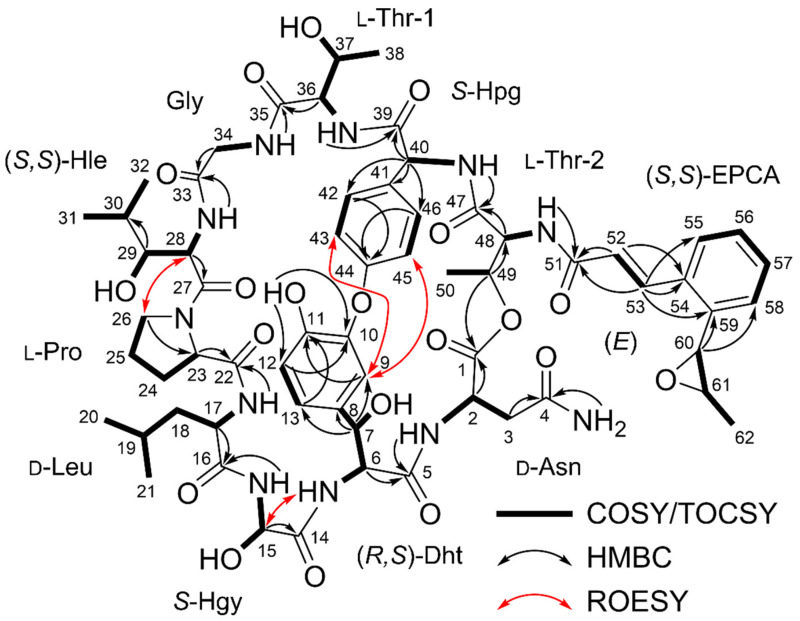
Key COSY/TOCSY, HMBC, and ROESY correlations of epoxinnamide (**1**). Dht = 3,*β*-dihydroxytyrosine, Hgy = *α*-hydroxyglycine, Hle = *β*-hydroxyleucine, Hpg = hydroxyphenylglycine, EPCA = *O*-1,2-epoxypropyl cinnamic acid. COSY = correlation spectroscopy, TOCSY = total correlation spectroscopy, HMBC = heteronuclear multiple bond correlation, ROESY = rotating-frame Overhauser effect spectroscopy.

COSY and TOCSY data showed that H-36, H-37, H_3_-38, 36-NH, and 37-OH (*δ*_H_ 4.44, 4.40, 1.08, 8.16, and 4.52) belong to one spin system, which was elucidated as another threonine unit (Thr-1). Another spin system was identified from 28-NH (*δ*_H_ 7.69) to two methyl groups (H_3_-31 (*δ*_H_ 0.72) and H_3_-32 (*δ*_H_ 0.74)) through H-28, H-29, and H-30 (*δ*_H_ 4.59, 3.22, and 1.74). H-29 also displayed COSY correlations with exchangeable proton 29-OH (*δ*_H_ 5.09). C-27 (*δ*_C_ 170.1) carbonyl carbon was attached to H-28 by H-28/C-27 HMBC correlation, finally elucidating a *β*-hydroxyleucine (Hle) residue (Figure 2).

Olefinic protons H-52 and H-53 (*δ*_H_ 6.94 and 8.11; ^3^*J*_H52H53_ = 15.5 Hz) have a COSY correlation with each other and HMBC correlations to carbonyl carbon C-51 (*δ*_C_ 169.2) and aromatic carbon C-53 (*δ*_C_ 134.1). H-53 also showed HMBC correlations with C-55 and C-59 (*δ*_C_ 126.9 and 136.74). COSY correlations of H-55, H-56, H-57, and H-58 (*δ*_H_ 7.42, 7.00, 7.33, and 7.09) and their coupling constants (7.5 Hz) enabled the construction of an *ortho*-substituted six-membered aromatic ring. H-60 (*δ*_H_ 4.07) is bound to a heteroatom-bearing carbon correlated with C-58 (*δ*_C_ 124.4) and C-59 in the HMBC spectrum. H-60, H-61 (*δ*_H_ 2.97), and H_3_-62 (*δ*_H_ 1.37) were identified to belong to a single spin system attached to C-59 based on COSY/TOCSY correlations. Moreover, no more exchangeable proton was available in the data, and the chemical shifts of H-61, H-62, C-61 (*δ*_C_ 58.9), and C-62 (*δ*_C_ 17.6) deduced the presence of the 1,2-propylene epoxide group. Thus, the last substructure of **1** was identified as an *o*-1,2-epoxypropyl cinnamic acid (EPCA) (Figure 2), which was once reported in nyuzenamide C. However, the ^1^H and ^13^C chemical shifts of the EPCA unit in **1** were different from those in nyuzenamide C: the chemical shifts of H-62 and C-52 deviated from those of the corresponding ^1^H and ^13^C atoms in EPCA of nyuzenamide C by 0.83 and 2.6 ppm, respectively.

The epoxinnamide (**1**) constituted six proteinogenic amino acids, four unusual amino acids, and an acyl chain, *o*-1,2-epoxypropyl cinnamic acid. By analysis of HMBC, correlations of *α*-amino protons and amide protons with carbonyl carbons assembled the subunits of **1** to Asn-Dht, Hgy-Leu-Pro, and Hle-Gly-Thr-1-Hpg-Thr-2-EPCA. These partial structures were further connected by analyzing rotating-frame Overhauser effect spectroscopy (ROESY) data (Figure S11). Dht and Hgy were tethered by a ROESY correlation between 6-NH and H-15, constructing the Asn-Dht-Hgy-Leu-Pro sequence. Similarly, Pro and Hle were connected based on H_2_-26 (δ_H_ 3.72/3.52)/H-28 ROESY correlation. In addition, the *β*-proton of Thr-2 (H-49 (*δ*_H_ 5.19)) showed an HMBC correlation to carbonyl carbon C-1 of Asn, thus elucidating the structure of epoxinnamide (**1**) as a cyclic deca-depsipeptide with an EPCA acyl chain.

However, 1 double-bond equivalent out of 29 unsaturations calculated based on the molecular formula was not explained by 12 carbonyl groups (12), 3 aromatic rings (12), 1 olefinic double bond (1), 1 epoxide (1), 1 proline ring (1), and 1 macrocyclic ring (1), indicating that epoxinnamide (**1**) requires one additional ring. Then, the open positions in the structure were only at the oxygen atoms of C-10 and C-44 because no more phenolic protons were observed. H-9 displayed ROESY correlations with H-43 and H-45 in the Hpg moiety, inferring a diaryl ether linkage between the Dht and Hpg to provide the required last ring. Finally, the structure of the epoxinnamide (**1**) was determined as an epoxy cinnamic acid-bearing bicyclic deca-depsipeptide (Figure 2). 

Epoxinnamide (**1**) is structurally related to nyuzenamides A-C (Figures S2 and S7), as they also have a bicyclic deca-depsipeptide backbone tethered with an alkenyl cinnamic acid-derived acyl chain [16,21]. However, they have two different amino acids in their deca-peptide cycles: **1** possesses *β*-hydroxyleucine and threonine instead of *β*-hydroxyphenylalanine and valine incorporated in the nyuzenamides. The phylogenetic analysis of the epoxinnamide-producing strain (*Streptomyces* sp. OID44) and the nyuzenamide-producing strains (*Streptomyces* sp. N11-34 and DM14) revealed that these strains are phylogenetically distinct consistently with the structural difference of epoxinnamide and the nyuzenamides (Figure S12). The epoxy cinnamic acid moiety is very unique and has been extremely rarely reported in nature, with only nyuzenamide C and NC-1 as examples [12,21].

The absolute configurations of *α*-carbons in the amino acid units were partly determined by the advanced Marfey’s method [23]. Epoxinnamide (**1**) was hydrolyzed into free amino acids by hydrochloride (HCl) and derivatized with l-1-fluoro-2,4-dinitrophenyl-5-alanine amide (l-FDAA) and d-FDAA. The FDAA derivatives were analyzed by liquid chromatography/mass spectrometry (LC/MS). According to the elution orders of the l and d-FDAA derivatives, the amino acid residues of **1** were established as d-Asn (detected as aspartic acid derivatives after acid hydrolysis), d-Leu, l-Pro, and l-Thr (Figure S13, Table S2). Despite the presence of two Thr units in **1**, only a single peak was detected in the LC/MS chromatogram of the FDAA derivatives. These results revealed that both Thr-1 and Thr-2 possess an l configuration. To determine the stereochemistry of the *β*-position in the threonines, authentic l-Thr and l-*allo*-Thr were derivatized with l and d-FDAA, and the derivatives were analyzed by LC/MS. Both Thr units in **1** were identified as l-Thr (Figure S14). In the case of the Hle units, the l-FDAA derivative was eluted earlier than the d-FDAA derivative. According to the previously reported analysis of Hle, this result assigned the stereochemistry of its *α*-position as an *S* configuration [24]. However, unusual amino acid units, Dht, Hgy, and Hpg, were not detected in the LC/MS analysis of Marfey’s products. Therefore, these units required further spectroscopic and genomic analysis for stereochemistry.

**Figure 3:**
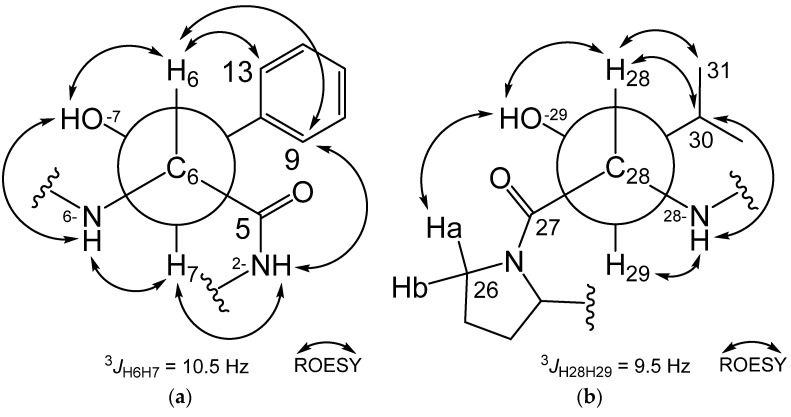
Vicinal proton–proton coupling constants (^3^*J*_HH_) and ROESY correlations in (**a**) 3*,β*-dihydroxytyrosine, and (**b**) *β*-hydroxyleucine.

The relative configurations of the *α*- and *β*-positions in the Dht and Hle units were determined through analysis of vicinal proton–proton coupling constants (^3^*J*_HH_) and key ROESY correlations (Figure 3). Analogously to nyuzenamide C, vicinal protons H-6 and H-7 showed a large coupling constant (^3^*J*_H6H7_ = 10.5 Hz) [21]. ROESY correlations in Dht unit revealed that **1** has the identical relative configuration of Dht with nyuzenamide C, 6R* and 7S* (Figure 3a). H-29 and H-30 showed a large coupling constant (^3^*J*_H29H30_ = 9.5 Hz), establishing their *anti*-relationship. H-28/29-OH, H-28/H-30, H-28/H_3_-31, 29-OH/H-26a, 28-NH/H-29, and 28-NH/H-30 ROESY correlations could be explained only with the rotamer shown in Figure 3b, assigning (2S*,3S*)-Hle (*erythro*-*β*-hydroxyleucine). Based on the determined absolute configuration of the *α*-carbon of the unit (28*S*), an *S* configuration was deduced for C-29.

To further support the absolute configuration of Hle units, the NMR data of previously reported Hle-bearing natural products were scrutinized in detail (Figure S15). The (2*S*,3*S*)-Hle units in epoxinnamide (**1**), telomycin [25], and dentigerumycin D [26] showed large coupling constants (^3^*J*_H*α*H*β*_ = 9.5–10.0 Hz) between their *α* and *β* protons. Esterified (2*S*,3*S*)-Hle-containing compounds, mollemycin [27] and muramycin B_1_ [28], also have large coupling constants (^3^*J*_H*α*H*β*_ = 10.3 and 9.2 Hz, respectively). In addition, (2*R*,3*R*)-Hle units in (−)-ternatin [29] and actinoramide A [30] also showed large coupling constants (^3^*J*_H*α*H*β*_ = 9.4 and 8.8 Hz, respectively). In contrast, (2*S*,3*R*)-Hle residues in sameuramide [31] and YM-254890 [32] exhibited small coupling constants (^3^*J*_H*α*H*β*_ 2.0 and 2.0 Hz, respectively). In addition, (2*R*,3*S*)-Hle in JBIR-78 [33], skyllamycin A [34], and laxaphycin B [35] possessed small coupling constants (^3^*J*_H*α*H*β*_ = 2.0, 1.9, and 2.0 Hz). By considering the listed examples, (2*S*,3*S*)- and (2*R*,3*R*)-Hle (otherwise called *erythro*-*β*-hydroxyleucine) in natural products have large ^3^*J*_H*α*H*β*_ (≥7 Hz), while (2*S*,3*R*)- and (2*R*,3*S*)-*β*-Hle (otherwise called *threo*-*β*-hydroxyleucine) have small ^3^*J*_H*α*H*β*_ (≤3 Hz). These observations indicated that the relative configuration of *β*-Hle could be predicted empirically based on the magnitude of their ^3^*J*_H*α*H*β*_ values.

The *trans*-coupling constant between H-52 and H-53 (15.5 Hz) is assigned 52*E* geometry. The small coupling constant of between the epoxide ring protons (^3^*J*_H60H61_ = 2.0 Hz) established that the epoxide ring is in a *trans* form (60S* and 61S*) [36,37]. To establish the absolute configuration of the epoxide part, conformational search and DP4 calculation were applied to the EPCA moiety. By comparing the experimental data and the calculated ^13^C/^1^H chemical shifts for **1a** (60*R* and 61*R*) and **1b** (60*S* and 61*S*) (Table S3), the DP4 calculation analysis deduced 60*S* and 61*S* (**1b**) with 99.7% probability (Figure S16). Therefore, the absolute configurations of EPCA moiety were proposed as *o*-(1*S*,2*S*)-epoxypropyl cinnamic acid.

**Figure 4:**
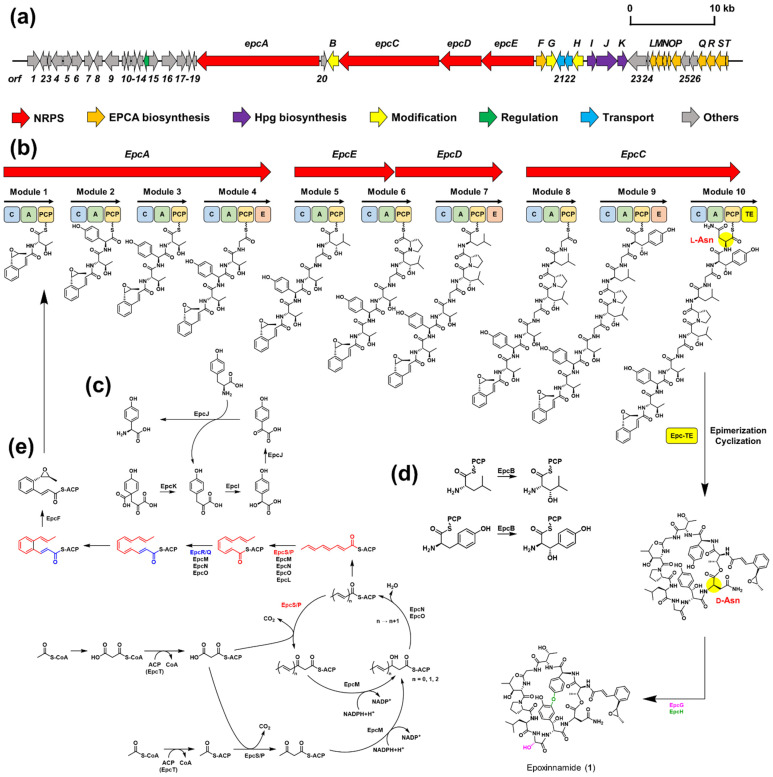
Putative biosynthesis of epoxinnamide (**1**). (**a**) Biosynthetic gene cluster (BGC) of **1**, (**b**) NRPS biosynthetic pathway of **1**, (**c**) biosynthetic pathway of l-hydroxyphenylglycine (l-Hpg), (**d**) biosynthesis of (2*S*,3*S*)-*β*-hydroxyleucine (Hle) and (2*R*,3*S*)-3*,β*-dihydroxytyrosine (Dht), (**e**) biosynthesis of *O*-(1*S*,2*S*)-epoxypropyl cinnamic acid (EPCA). Wavy bonds at the PCP domains represent the 4′-phosphopantetheine groups. NRPS = nonribosomal peptide synthetase, C = condensation domain, A = adenylation domain, PCP = peptidyl carrier protein domain, E = epimerization domain, TE = thioesterase domain, ACP = acyl carrier protein, NADP = nicotinamide adenine dinucleotide phosphate.

The absolute configurations of the Dht, Hgy, and Hpg units were not yet determined with our spectroscopic analysis. Therefore, whole-genome sequencing of epoxinnamide-producing *Streptomyces* sp. OID44 was performed. Based on the analysis of the whole-genome sequence by utilizing antiSMASH 6.0 [38] (Table S4), a putative biosynthetic gene cluster (BGC) of epoxinnamide (**1**) was identified. The BGC of **1** showed a 57% similarity with BGC of atratumycin [13] and exhibited high homology (74%) to the BGC of nyuzenamide C [21], which is structurally most similar to epoxinnamide (Figure S7). The BGC of epoxinnamide (**1**) consists of four modular, non-ribosomal peptide synthetase (NRPS)-encoding genes, and biosynthetic genes of unusual amino acids units and epoxypropyl cinnamic acid (Figure 4a and Table S5). The NRPSs (EpcA, EpcC, EpcD, and EpcE) were composed of 10 modules corresponding to the incorporation of the 10 amino acid units in **1**. EpcA encodes four modules containing 13 domains, which consist of condensation (C), adenylation (A), peptidyl carrier protein (PCP), and epimerization (E) domains (C-A-PCP, C-A-PCP, C-A-PCP, and C-A-PCP-E). EpcC encodes three modules containing 11 domains with C, A, PCP, E, and thioesterase (TE) domain (C-A-PCP, C-A-PCP-E, C-A-PCP-TE). Next, EpcD encodes two modules with five domains (PCP, and C-A-PCP-E), and EpcE encodes two modules with five domains (C-A-PCP, and C-A) (Figure 4b).

The E domain in module 7 supported the d-Leu determined by the advanced Marfey’s method. Module 9, incorporating *β*-hydroxytyrosine (later converted to 3*,β*-dihydroxytyrosine, Dht), contained an E domain, indicating *R*-configuration of its *α*-carbon. The relative configuration of Dht was identified as a *threo* form, and the absolute configurations of this unit were determined as (2*R*,3*S*)-3*,β*-dihydroxytyrosine. Therefore, the absolute configurations of C-6 and C-7 were deduced as being *R* and *S*, respectively. However, despite the absence of E domain in module 10, the chemical analysis of Marfey’s products showed that the Asn was in d form. This contradictory result could be explained by the special function of the thioesterase domain in module 10, Epc-TE in the BGC of epoxinnamide (**1**), similarly to Dml-TE in the BGC of nyuzenamide C (Figure S17). For detailed comparative analysis, the structures of these thioesterase domains were predicted by Phyre2 [39]. These two thioesterase domains showed a 60% of sequence identity to Skyxy-TE, which catalyzes both epimerization and macrocyclization in biosynthesis of skyllamycin [40]. Several amino acid residues exist (Pro31, Ala32, Trp96, Ser97, Leu98, Asp124, Gln125, Pro139, Phe202, and His254) that play important roles in the function of Skyxy-TE (Figure S18). Among them, Asn125 of Skyxy-TE played key roles in the epimerization process, whereas Phe202 of Skyxy-TE is crucial in macrocyclization. All of these active site residues except Gln125 were conserved in the Epc-TE sequence. Gln125 was replaced with Glu125, which is structurally analogous to Gln125. The side-chain amide group of Gln125 forms hydrogen bonds with adjacent main-chain amide oxygens in Skyxy-TE [40]. Since glutamic acid has a similar size to glutamine and also has a hydrogen atom capable of hydrogen bonding, we could predict that Epc-TE would still have both epimerizing and cyclizing functions, analogously to Skyxy-TE (Figure S19). Although Dml-TE has one more conservative replacement, Ala32 to Val32, it is thought that the bifunctional activity of TE would be maintained (Figure S19). Interestingly, the thioesterase domain in BGC of atratumycin (Atr-TE) is also thought to have these dual functions. Despite a deficiency of an E domain in the last module, the last amino acid unit, tyrosine, was identified as being in d form in atratumycin (Figure S17) [13]. Atr-TE has a slight similarity to Skyxy-TE (identity 50%), but the Gln125 and seven more active site residues have been conserved in its sequence (Figures S18 and S19). These unique bifunctional thioesterase domains, which catalyze both epimerization and macrocyclization, were mainly found in BGC of the cinnamoyl moiety-containing compounds. On the other hand, in the case of telomycin without cinnamoyl moiety, its thioesterase domain (Tel-TE) showed low similarity with Skyxy-TE (identity 42%). It has non-conservative replacements at the important active site residues, Gln125 and Phe202 to Thr124 and Met201, so the last amino acid unit was in the l form (Figures S17–S19). Through these observations, we could conclude that Glx125 (Gln or Glu) is an important residue in the epimerization function. Thus, it would be possible to commonly identify the homologous active sites of the bifunctional thioesterase domains through amino acid sequence analysis and protein structure predictions of Epc-TE, Dml-TE, and Atr-TE [13,21,39,40].

The proposed functions of EpcI, EpcJ, and EpcK were related to the biosynthesis of Hpg units. The Hpg units were found in the structures of ramoplanin, enduracidin, calcium-dependent antibiotics and many glycopeptide antibiotics [41]. Biosynthesis of the Hpg proceeds through four steps, beginning with prephenate [42]. EpcK showed 62% sequence identity with prephenate dehydrogenase in the BGC of enduracidin [43], and it was expected to catalyze the reaction from prephenate to 4-hydroxyphenylpyruvate. EpcI has a 53% shared sequence identity with 4-hydroxymandelate synthase (HmaS) in the BGC of calcium-dependent antibiotics [44], and this could propose the function of EpcI in the synthesis of l-4-hydroxymandelate. EpcJ showed high homology (63% identity) with hydroxyphenylglycine aminotransferase (HpgT)/hydroxymandelate oxidase (HmaO) fusion protein in the BGC of enduracidin [43]. It catalyzes reactions from l-4-hydroxymandelate to 4-hydroxybenzoylformate (by the HmaO) and from 4-hydroxybenzoylformate to l-4-hydroxyphenylglycine (by HpgT). HpgT used l-tyrosine (Tyr) as an amino-donor co-substrate and converted l-Tyr into 4-hydroxyphenylpyruvate [41]. In summary, three enzymes (EpcI, EpcJ, and EpcK) could be involved in synthesizing the *S*-hydroxyphenylglycine from prephenate and l-Tyr. Due to the absence of an E domain in module 2, the Hpg unit in the epoxinnamide (**1**) was proposed as being in *S* form (40*S* configuration) (Figure 4c).

Detailed analysis of the BGC of epoxinnamide also supported the stereochemistry of *β*-carbons in Hle and Dht units. EpcB showed a highly similar sequence identity to the cytochrome P450 monooxygenases DmlC, Sky32, Tem23, and Atr27 (94%, 66%, 61%, and 55%, respectively) in NRPS biosynthesis (Figure S20). DmlC catalyzes the *β*-hydroxylation of phenylalanine and tyrosine residues in the biosynthesis of nyuzenamide C [21]. Sky32 catalyzes the *β*-hydroxylation of three PCP domain-bound amino acids (phenylalanine, *O*-methyltyrosine, and leucine) in the skyllamycins, and it showed *S*-stereoselective hydroxylation activity (Figure S17) [45]. Tem23 is the cytochrome P450 monooxygenase responsible for the *β*-hydroxylation of leucine in telomycin [46]. Moreover, in the BGC of atratumycin, cytochrome P450 monooxygenase Atr27 catalyzes the *β*-hydroxylation of phenylalanine unit in PCP domain-dependent manner [13]. All of these enzymes constructed the *S*-hydroxylated *β*-amino acids (Figure S17). Through these observations, the function of EpcB was identified as *β*-hydroxylase, which catalyzes the *S*-stereoselective hydroxylation of leucine and tyrosine in PCP domain-dependent manners (Figure 4d). Our phylogenetic analysis of cytochrome P450 *β*-hydroxylase indicated that EpcB and DmlC are closely related to *S*-stereoselective *β*-hydroxylase Sky32 (Figure S21 and Table S6), which additionally supported the chemically determined *S* configuration at the *β*-position of 3*,β*-dihydroxytyrosine and *β*-hydroxyleucine in **1**.

The EPCA moiety was biosynthetically intriguing. Recently, it was found that highly reducing (HR) type II polyketide synthases (PKSs) catalyze the biosynthesis of the alkenyl cinnamoyl moieties in youssoufene and kitacinnamycin [47,48]. Type II PKSs are comprised of a minimal set of iteratively used enzymes, acyl carrier protein (ACP), ketosynthase (KS), and chain length factor (CLF) [49]. Comparing the BGCs of epoxinnamide, youssoufene, and kitacinnamycin, we found 10 enzymes (EpcF and EpcL-EpcT) possibly related to the biosynthesis of EPCA (Figure 4e). Transacylation of the precursor, malonyl-CoA to malonyl-ACP, could be performed by ACP homologue EpcT. In type II PKS, transacylation of the malonyl-CoA unit to ACP is performed by malonyl-CoA:holo-ACP transacylase (MCAT) or self-malonylation [50]. A recent study revealed that Kcn4, the ACP in biosynthetic pathway of kitacinnamycin, has the self-acylation ability [48]. Because EpcT showed high homology with Kcn4 (83% identity), EpcT was proposed to have the self-acylation activity. Through phylogenetic analysis of *β*-ketoacyl-ACP synthases, EpcR and EpcS are predicted as being KSs, whereas EpcP and EpcQ possibly function as CLFs (Figure S22). These enzymes were proposed to act in the form of KS/CLF complexes, EpcS/P and EpcR/Q. Heterodimer EpcS/P could catalyze the biosynthesis of *β*-ketoacyl-ACP. Next, the *β*-ketoacyl-ACP reductase homologue (EpcM) and *β*-ketoacyl-ACP dehydratase homologues (EpcN and EpcO) reduced the *β*-ketoacyl-ACP to enoyl-ACP intermediate. Thus, a C_8_-polyene intermediate could be constructed through these enzymatic reactions. The putative function of isomerase EpcL was predicted to be responsible for the formation of the *cis* configuration. This enzyme could catalyze the synthesis of (2*E*, 4*Z*, 6*E*, 8*E*)-C_10_ polyene with EpcS/P, EpcM, EpcN, and EpcO. After this reaction, another KS/CLF complex, EpcR/Q, was proposed to catalyze the elongation from C_10_-polyene to C_12_-polyene. 

In summary, EpcS/P was proposed to initiate the chain elongation, and EpcR/Q was proposed to synthesize the final C_12_-polyene intermediate. In the case of the kitacinnamycin and youssoufene, Kcn17-Kcn18-Kcn19 and YssX catalyze the 6*π*-electrocyclic ring closure of polyene to form a benzene ring [48]. However, a homologue of these enzymes was not found in the BGC of epoxinnamide (**1**); thus, the formation of the benzene ring in **1** requires further research. In the previous report, epoxidation of a cinnamoyl moiety was catalyzed by cytochrome P450 [14]. Thus, the epoxide ring formation in epoxinnamide (**1**) was predicted to be catalyzed by cytochrome P450 EpcF, which showed 36% identity with epoxidation-related cytochrome P450 SlgO1 [51].

The last unassigned stereogenic center was *α*-carbon in the Hgy unit. This unit was already found in skyllamycin and dolyemycin [52], secondary metabolites of *Streptomyces* spp. Both Hgy units in these compounds were identified as *S*-Hgy based on total synthesis [34] and X-ray crystallography, respectively. The Hgy unit was constructed by the *α*-hydroxylase Sky39 in skyllamycin [8]. EpcG in the BGC of epoxinnamide showed high homology with Sky39 (66% identity), and thus, the function of the EpcG was proposed as glycine *α*-hydroxylase. Because Sky39 catalyzes stereoselective hydroxylation of glycine to *S*-hydroxyglycine, it could be inferred that the homologous enzyme EpcG also generates *S*-hydroxyglycine, thus proposing the 15*S* configuration, which is similar to the nyuzenamide C (Figure S7).

Bicyclic diaryl ether linkages were found in the structures of glycopeptide antibiotics such as vancomycin, teicoplanin, and A47934. These ether linkages between two phenol rings were constructed by cytochrome P450 OxyB enzyme [53]. EpcH, cytochrome P450 in the BGC of epoxinnamide, showed 35% of sequence identity with OxyB in the BGC of teicoplanin [54]. In addition, it is similar to SlgO2 and TamI (51% and 48%), which catalyzes the formation of bicyclic ketal moieties [51,55]. Consequently, EpcH could be predicted to catalyze the formation of bicyclic diaryl ether linkage in epoxinnamide (**1**).

### 2.2. Bioactivity of Epoxinnamide *(**1**)*

**Figure 5:**
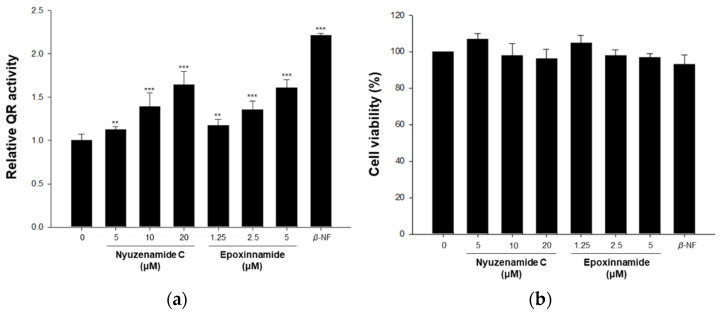
Induction of quinone reductase (QR) activity by nyuzenamide C and epoxinnamide (**1**) in Hepa1c1c cells. (**a**) Hepa1c1c7 cells were seeded in 24-well plates, incubated for 24 h, and treated with nyuzenamide C, epoxinnamide (**1**) and *β*-naphthoflavone (*β*-NF, 2 μM, a positive control) for an additional 24 h. QR activity was determined as described in the materials and methods section. (**b**) Cell viability was evaluated by crystal violet staining in Hepa1c1c7 cells. Stained cells were dissolved in 0.5% sodium dodecyl sulfate (SDS) in 50% ethanol solution, and absorbance was measured at 610 nm. All data are expressed as the mean values ± standard deviations (*n* = 3) and represent three separate experiments. ** *p* < 0.01, *** *p* < 0.001 compared to the control.

Quinone reductase (QR) is a major phase II enzyme that plays a significant role in detoxifying xenotoxicants. The induction of the phase II enzyme is considered to have an important chemoprevention effect on cancer [56]. In our previous study, nyuzenamide C, a cyclic peptide with a cinnamic acid moiety, enhanced QR activity [21]. Based on the structural similarity, the effect of epoxinnamide (**1**) on QR activity was evaluated together with nyuzenamide C. Epoxinnamide (**1**) and nyuzenamide C induced QR activity 1.60-fold and 1.12-fold at a test concentration of 5 μM, respectively, without cytotoxicity against the Hepa-1c1c7 murine hepatoma cell line. This indicates that the induction of QR activity by epoxinnamide (**1**) is stronger than that of nyuzenamide C (Figure 5).

**Figure 6:**
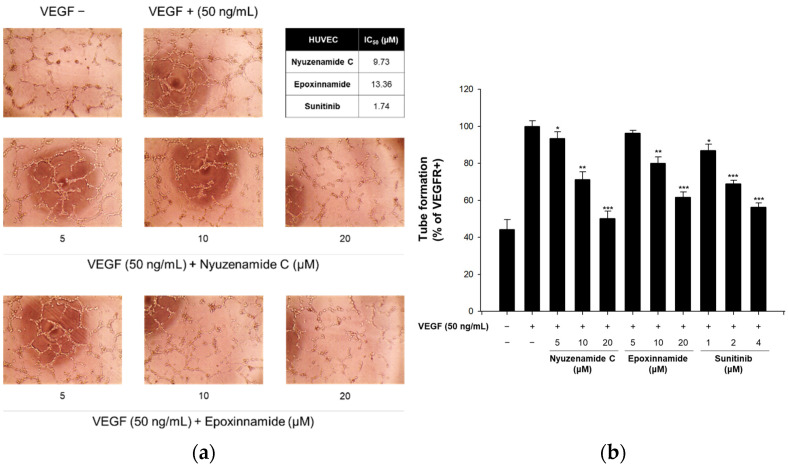
Effects of epoxinnamide (**1**) on the tube formation assay in human umbilical vein endothelial cells (HUVECs). (**a**) Photograph of the tube formation. (**b**) Quantified tube formation with a bar graph. HUVECs were seeded in Matrigel-coated 96-well plates and treated with vascular endothelial growth factor (VEGF, 50 ng/mL) and various concentrations of nyuzenamide C and epoxinnamide (**1**) for 6 h. Data are expressed as the mean values ± standard deviations of three separate experiments. * *p* < 0.05, ** *p* < 0.01, *** *p* < 0.001 compared to the vehicle-treated control.

Angiogenesis is the formation of new vessels from pre-existing blood vessels, which is important in wound healing and embryonic development. However, excessive angiogenesis is involved in various diseases, including retinopathy, cancer, and cerebral infarction [57]. Because nyuzenamide C previously exhibited antiangiogenic activity [21], epoxinnamide (**1**) was also evaluated in our angiogenesis assay using cultured human endothelial cells. As a result, epoxinnamide (**1**) effectively inhibited the tube formation in the vascular endothelial growth factor (VEGF)-induced endothelial cells with a median inhibitory concentration (IC_50_) value of 13.4 µM, which is milder than that of nyuzenamide C (IC_50_ = 9.73 μM) (Figure 6).

Although **1** and nyuzenamide C are structurally related by containing the EPCA moiety and eight amino acid units, they have two different amino acid residues. The incorporation of *β*-hydroxyleucine and threonine in **1** instead of *β*-hydroxyphenylalanine and valine in nyuzenamide C increased the QR-inducing activity. Still, it decreased the antiangiogenic effects, indicating a preliminary structure–activity relationship in this class of CCNPs.

## 3. Materials and Methods

### 3.1. General Experimental Procedures

Specific rotation was measured by a JASCO P-2000 polarimeter with a 10 mm cell at 20 °C. Ultraviolet (UV) data were acquired by an Applied Photophysics Chirascan-Plus circular dichroism spectrometer using a 1 mm UV cell. Infrared (IR) spectra were recorded by a JASCO Fourier transform/infrared spectrometer (FT/IR-4200). One-dimensional (1D) and two-dimensional (2D) nuclear magnetic resonance (NMR) spectra were obtained by a Bruker Avance III HD 800 MHz NMR spectrometer located at the College of Pharmacy, Seoul National University, Republic of Korea. Chemical shifts of all NMR spectra were referenced to the residual protonated solvent peaks of dimethyl sulfoxide (DMSO)-*d*_6_ (*δ*_H_ 2.50/*δ*_C_ 39.5). Liquid chromatography/mass spectrometry (LS/MS) data and low-resolution electrospray ionization mass spectrometry (LRESIMS) data were acquired using an Agilent Technologies 1200 series high-performance liquid chromatography (HPLC) coupled with an Agilent Technologies 6130 series single quadrupole ESIMS instrument. High-resolution ESIMS (HRESIMS) experiments were carried out on an AB Sciex 5600 quadrupole time-of-flight (QTOF) HRMS instrument at the National Instrumentation Center for Environmental Management (NICEM), Seoul National University, Republic of Korea.

### 3.2. Bacterial Isolation

A mud sample was collected from the intertidal mudflat in Oido (37.38545° N, 126.68576° E), Siheung, Gyeonggi-do, the Republic of Korea, on 1 April 2021. Then, 10 mL of sterilized deionized water was used to dilute the sample. A portion of the mixture (100 μL) was spread onto A4 medium (agar 18 g, sterilized deionized water 1 L), actinomycete isolation agar medium, Czapek-Dox medium, ISP medium 4, marine 2216 medium, modified K medium (yeast extract 3 g, d(+)-glucose 2 g, d(−)-mannitol 2 g, malt extract 5 g, soluble starch 5 g, soytone 5 g, calcium carbonate 1 g, sterilized deionized water 1 L), starched casein medium, YEME medium (yeast extract 4 g, d(+)-glucose 4 g, malt extract 10 g, sterilized deionized water 1 L), and YPM medium (yeast extract 2 g, d(−)-mannitol 4 g, peptone 2 g, sterilized deionized water 1 L). All of the media were made with agar 18 g, cycloheximide 100 mg, and sea salts 28 g. The agar plates were stored in an incubator (30 °C) for 20 days. A pure bacterial strain OID44 was isolated from ISP medium 4.

### 3.3. Phylogenetic Analysis of Streptomyces sp. OID44

To examine the phylogenetic position of epoxinnamide-producing strain OID44 and its relationship with nyuzenamide-producing strains N11-34 and DM14, 16S rRNA gene sequences of the strains were analyzed, together with those of the closely related type strains that were searched at the EzBioCloud database [58]. All sequences were aligned using SINA online aligner [59] and filtered by the positional variability filter for bacteria (“pos_var_ssuref:bacteria”), as implemented in ARB software. The aligned sequences were used for tree-building by MEGA11 based on the neighbor-joining method [60]. The epoxinnamide-producing strain OID44 was phylogenetically distinct from the nyuzenamide-producing strains, as revealed by different positions in the phylogenetic tree (Figure S12). Strain OID44 showed sequence similarities of 98.6% and 98.5% to strains N11-34 and DM14, respectively. The most closely related type strain of OID44 was *Streptomyces antimycoticus* NBRC 12839^T^, with only 1 bp difference, while both N11-34 and DM14 showed the highest sequence similarity to *Streptomyces hygroscopicus* subsp. *hygroscopicus* NBRC 13472^T^, with also only 1 bp difference (Figure S12).

### 3.4. Large-Scale Culture and Extraction

The OID44 strain was inoculated into 50 mL of YEME saline (YEME medium with 28 g/L of sea salts) liquid medium in a 125 mL Erlenmeyer flask and incubated in a 30 °C, 180 rpm rotary shaker. After 3 days of cultivation, 5 mL of the culture was transferred to 250 mL of YEME saline liquid medium in a 500 mL baffled Erlenmeyer flask. The liquid culture was incubated in a 30 °C, 170 rpm rotary shaker for 3 days. After this, 25 mL of the culture was transferred to 1 L of YEME saline liquid medium in 2.5 L Ultra Yield^®^ flasks (40 flasks, total 40 L culture) and incubated in a 30 °C, 160 rpm rotary shaker. After 6 days, the whole culture was extracted with ethyl acetate (EtOAc) using a separation funnel. The mixture was separated into two layers, and the water layer was extracted with EtOAc by the same procedure to maximize the yield of bacterial secondary metabolites. After the separation of the EtOAc layer from the water layer, residual water in the EtOAc was removed by anhydrous sodium sulfate. A rotary evaporator concentrated the EtOAc extract in vacuo in the dark, and 5 g of the dried extract was acquired.

### 3.5. Purification of Epoxinnamide *(**1**)*

The crude extract of the OID44 strain was adsorbed in 2.5 g of Celite and loaded onto a reverse-phase open column (60 × 40 mm, YMC*GEL ODS-A, 12 nm, S-75 μm). The adsorbed extract was fractionated by 200 mL of stepwise methanol/water (MeOH/H_2_O) solution (20, 40, 60, 80, and 100%). Aliquots (20 μL) of the fractions were diluted with 180 μL of methanol and analyzed using LC/MS under a gradient system (flow rate: 0.7 mL/min; UV detection: 210, 230, 254, 280, and 360 nm; acetonitrile/water (CH_3_CN/H_2_O with 0.1% formic acid) 10% to 100% over 20 min) with a Phenomenex column (Luna^®^, 5μm, C_18_(2), 100 Å, 100 × 4.6 mm). Epoxinnamide (**1**) was observed in the 60% and 80% MeOH/H_2_O fractions. The fractions were concentrated and dissolved in 1 mL of MeOH. The solution was further fractionated by Sephadex LH-20 column chromatography using MeOH as an eluent. The epoxinnamide fraction was concentrated and dissolved in 1 mL of MeOH and filtered using a syringe filter (FILTSTAR syringe filter 13 mm, hydrophilic PTFE 0.22 μm). The filtrate was injected into a semi-preparative reversed-phase HPLC system (YMC-Pack ODS-A, 250 × 10 mm, S-5 μm, 12 nm, gradient solvent system: CH_3_CN/H_2_O 40% to 75% over 40 min, UV detection 210 nm, flow rate 2.0 mL/min). Epoxinnamide (**1**) was eluted at a retention time of 20 min and then quickly extracted by EtOAc. Finally, 10 mg of the epoxinnamide (**1**) was acquired. All the procedures were carried out under minimal exposure to light.

*Epoxinnamide* (**1**): white amorphous powder, ${\left[\alpha \right]}_{D}^{20}$ + 35 (c = 0.1, MeOH); UV (MeOH) λ_max_ (log ε) 280 nm (2.97); IR (neat) ν_max_ 3285, 1658, 1538 cm^−1^; NMR data in DMSO-*d*_6_, Table 1; HRESIMS [M + H]^+^ *m*/*z* 1298.5611 (calcd for C_62_H_79_N_11_O_20_, 1298.5576).

### 3.6. Determination of the Absolute Configuration at the α- and β-Carbons of the Amino Acid Units

First, 1 mg of epoxinnamide (**1**) was hydrolyzed by using 1 mL of 6 N HCl at 115 °C for 1 h with stirring. The hydrolysate was rapidly cooled in an ice bath for 5 min and quenched with 6 mL of 1 N NaHCO_3_. The mixture was dried in vacuo for 24 h and divided into two vials. Each hydrolysate was dissolved in 200 μL of 1 N NaHCO_3_, and 100 μL of l-1-fluoro-2,4-dinitrophenyl-5-alanine amide (l-FDAA) and d-FDAA in acetone (10 mg/mL) was added to each vial. Both vials were heated at 80 °C for 5 min with stirring and quenched with 100 μL of 2 N HCl. The reaction products were lyophilized for 24 h and re-dissolved in 500 μL of MeOH. The solutions were filtered with a syringe filter (FILTSTAR syringe filter 13 mm, hydrophilic PTFE 0.22 μm). The filtrates were analyzed with LC/MS using a gradient solvent system (Phenomenex, Luna^®^, 5 μm, C_18_(2), 100 Å, 100 × 4.6 mm; flow rate: 0.7 mL/min; UV detection: 340 nm; CH_3_CN/H_2_O with 0.1% formic acid 10% to 60% over 50 min). The retention times of the FDAA derivatives were analyzed by negative mass ion extraction.

### 3.7. Conformation Search and DP4 Analysis

A conformational search of the epoxide-bearing partial structure of **1** (Figure S16) was carried out using a mixed sampling method of torsional/low-mode using MacroModel (version 9.9, Schrödinger LLC, New York, NY, USA) in the Maestro suite (version 9.9, Schrödinger LLC). A total of 39 diastereomeric conformers were acquired under 10 kJ/mol relative potential energy using the Merck molecular force field [61]. The shielding constants of the optimized conformer were calculated according to Equation (1) [62], where $\sigma^{x}$ is the Boltzmann-averaged shielding constant for nucleus x, $\sigma_{i}^{x}$ is the shielding constant for nucleus x in conformer i, and $p_{i}$ is the probability of conformer i in the Boltzmann distribution.

(1) $$\sigma^{x}=\frac{\sum_{i}\sigma_{i}^{x}p_{i}}{\sum_{i}p_{i}}$$

Chemical shifts were calculated according to Equation (2) [62], where $\delta_{calc}^{x}$ is the calculated chemical shifts for nucleus x, and $\sigma^{o}$ is the shielding constant for the proton and carbon nuclei calculated at the TD-DFT-B3LYP/6-31G+(d,p) level in the gas phase using the TmoleX 4.3.2 software [63].

(2) $$\delta_{calc}^{x}=\frac{\sigma^{o}-\sigma^{x}}{1-\sigma^{o}/10^{6}}$$

### 3.8. Genome Analysis of Streptomyces sp. OID44

Whole-genome analysis of *Streptomyces* sp. OID44 was obtained from CJ Bioscience, Inc. (Seoul, Korea) [58] using a Pacbio Sequel system (Pacific Biosciences, Menlo Park, CA, USA) [64]. The sequencing data were assembled with SMRT Link using the Microbial Assembly Protocol (Pacific Biosciences, Menlo Park, CA, USA) [65]. The protein-coding sequences (CDSs) were predicted using Prodigal 2.6.2. The CDSs were annotated with references to EggNOG 4.5, Swissprot, KEGG, and SEED. A biosynthetic gene cluster (BGC) of epoxinnamide was identified using antiSMASH software (version 6.0) [38], and it was deposited in GenBank under accession number ON243978. Sequence alignments were performed with Geneious Alignment (alignment type: global alignment (Needleman–Wunsch), global alignment with free end gaps, cost matrix: Blosum 62), and a phylogenetic tree was built with Geneious Tree Builder (Jukes–Cantor, neighbor-joining) using Geneious Prime 2022.1.1 software (Biomatters Ltd., Auckland, New Zealand, https://www.geneious.com, accessed on 12 July 2022). The protein structures of thioesterase domains were predicted by Phyre2 [39]. Structure visualizations of the proteins were performed with UCSF ChimeraX 1.3 [66].

### 3.9. Quinone Reductase Assay

The QR activity was determined using Hepa-1c1c7 murine hepatoma cells (American Type Culture Collection, Manassas, VA, USA), as described previously [67]. Hepa-1c1c7 cells were cultured in minimum essential medium-*α* with 10% fetal bovine serum and 1% antibiotic–antimycotic (100 units/mL penicillin G sodium, 100 μM streptomycin, and 250 ng/mL amphotericin B). The cells (4 × 10^4^ cells/mL) were seeded in a 24-well plate. After incubation for 24 h, each well was treated with various concentrations of nyuzenamide C and epoxinnamide (**1**). *β*-Naphthoflavone (2 μM) was used as a positive control. After treatment for 24 h, the media were removed, and the cells were lysed with a buffer (250 μL) containing 10 mM Tris-HCl (pH 8.0), 140 mM NaCl, 15 mM MgCl_2_, and 0.5% NP-40 (IGEPAL CA-630) (Sigma-Aldrich, St. Louis, MI, USA). After incubation for 10 min, the complete reaction mixture (1 mL), containing 12.5 mM Tris-HCl (pH 7.4), 0.67 mg/mL bovine serum albumin, 0.01% Tween-20, 50 μM flavin adenine dinucleotide, 1 mM glucose-6-phosphate, 2 U/mL glucose-6-phosphate dehydrogenase, 30 μM nicotinamide adenine dinucleotide phosphate (NADP), 50 μg/mL 3-(4,5-dimethylthiazo-2-yl)-2,5-diphenyltetrazolium bromide (MTT), and 50 μM menadione, was added to each well. When the colorimetric reaction was complete, the rate of the NADPH-dependent menadiol-mediated reduction of MTT was measured at 610 nm. A cytotoxicity evaluation was performed by crystal violet staining of the identical set of test plates, with the measurement at 610 nm. The activity of quinone reductase (nmol/min/mg of protein) was calculated as follows: (change in the absorbance of MTT per minute)/(the absorbance of crystal violet) × 3345 nmol/mg, where 3345 is the ratio of the proportionality constant determined for crystal violet. The relative QR activity was normalized by using controls.

### 3.10. In Vitro Capillary Tube Formation Assay

Matrigel (70 μL/well) was used to coat a 96-well plate, which was seeded in an incubator for 30 min at 37 °C. Human umbilical vein endothelial cells (HUVECs) (1.3 × 10^4^ cells/well) were mixed with various concentrations of nyuzenamide C and epoxinnamide (**1**), then seeded onto each well of the Matrigel-coated 96-well plate. After the seeding, the plate was incubated at 37 °C in a 5% CO_2_ atmosphere for 6 h, and endothelial cell tubular structures were formed on each well. An inverted microscope (Olympus Optical Co., Ltd., Tokyo, Japan) was operated to visualize and photograph the formations of the tubular structure. These images were measured with the Angiogenesis Analyzer of the ImageJ program. The total segment lengths of the structures were used to calculate the tube-formation activity and to evaluate the degree of tube formation [68].

## 4. Conclusions

A new cinnamoyl-containing nonribosomal peptide (CCNP), epoxinnamide (**1**), was discovered from intertidal mudflat-derived *Streptomyces* sp. OID44. The structure of **1** was elucidated as a bicyclic deca-depsipeptide with an epoxypropyl cinnamic acid (EPCA) acyl chain via the analysis of HRESIMS and NMR data. The absolute configurations of *α*- and *β*-amino carbons were determined with a combination of an advanced Marfey’s method, ^3^*J*_HH_ and ROESY analysis, and bioinformatic analysis. The stereochemistry of the EPCA moiety was established through a conformational search and DP4 calculation. The NRPS biosynthetic pathway of the epoxinnamide (**1**) was proposed through the whole-genome sequencing of *Streptomyces* sp. OID44. The BGC of **1** contains a unique bifunctional thioesterase domain, which catalyzes both epimerization and macrocyclization, and highly reducing (HR) type II PKSs, which construct its cinnamoyl acyl chain. The structure of epoxinnamide (**1**) is most closely related to that of nyuzenamide C in that they share the bicyclic deca-depsipeptide scaffold with the same EPCA acyl chain. However, their amino acid compositions are different because, in epoxinnamide, *β*-hydroxyleucine and threonine replaced the *β*-hydroxyphenylalanine and valine in nyuzenamide C. Epoxinnamide (**1**) showed significantly higher quinone reductase-inducing activity but slightly lower antiangiogenesis activity than nyuzenamide C. Our discovery of a new member of the structurally unique and biologically active CCNP family from a marine-derived actinobacterial strain also highlights the importance of actinomycetes in marine habitats in the search for new bioactive compounds.

## Figures and Tables

**Table 1:** NMR spectroscopic data (800 MHz, dimethyl sulfoxide (DMSO)-*d*_6_) for epoxinnamide (**1**).

Position	*δ*_C_, Type	*δ*_H_ (*J* in Hz)	Position	*δ*_C_, Type	*δ*_H_ (*J* in Hz)
d-Asn			28-NH		7.69 ^a^, d (9.5)
1	169.6, C		29	74.4, CH	3.22, ddd (9.5, 2.0, 2.0)
2	49.0, CH	3.99, ddd (9.5, 7.0, 3.5)	29-OH		5.09, br d (2.0)
2-NH		8.23, d (7.0)	30	27.9, CH	1.74, m
3a	35.1, CH_2_	2.65, dd (15.5, 3.5)	31	14.4, CH_3_	0.72, d (7.0)
3b		2.57, dd (15.5, 9.5)	32	20.2, CH_3_	0.74, d (7.0)
4	170.64, C		Gly		
4-NH_2_a		7.43 ^a^, br s	33	168.4, C	
4-NH_2_b		7.03, br s	34a	42.60, CH_2_	4.18, dd (17.5, 8.5)
(*R*,*S*)-Dht			34b		3.39, dd (17.5, 5.0)
5	170.63, C		34-NH		7.68 ^a^, dd (8.5, 5.0)
6	63.8, CH	3.49, dd (10.5, 3.5)	l-Thr-1		
6-NH		8.14 ^a^, br s	35	170.0, C	
7	69.5, CH	4.69, dd (10.5, 6.5)	36	57.7, CH	4.44, dd (10.0, 3.5)
7-OH		6.15, d (6.5)	36-NH		8.16, d (10.0)
8	132.3, C		37	65.8, CH	4.40, m
9	120.1, CH	6.27, d (2.0)	37-OH		4.52, d (7.0)
10	147.3, C		38	20.6, CH_3_	1.08, d (6.5)
11	147.4, C		*S*-Hpg		
11-OH		9.40, s	39	169.1, C	
12	116.1, CH	6.87, d (8.5)	40	60.1, CH	4.83, d (3.0)
13	120.2, CH	7.08 ^a^, dd (8.5, 2.0)	40-NH		7.55, d (3.0)
*S*-Hgy			41	131.6, C	
14	170.9, C		42	128.5, CH	7.07 ^a^, dd (8.5, 2.0)
15	71.3, CH	5.79, dd (9.5, 5.5)	43	123.6, CH	6.42, dd (8.5, 2.5)
15-OH		7.17 ^a^, br s	44	159.6, C	
15-NH		9.07, d (9.5)	45	122.3, CH	7.18, dd (8.0, 2.5)
d-Leu			46	131.3, CH	7.37, dd (8.0, 2.0)
16	171.5, C		l-Thr-2		
17	50.3, CH	4.64, td (9.5, 5.0)	47	173.3, C	
17-NH		8.44, d (9.5)	48	61.0, CH	5.03, d (4.5)
18a	42.58, CH_2_	1.45 ^a^, m	48-NH		8.96, d (4.5)
18b		1.31, m	49	68.7, CH	5.19, q (7.0)
19	24.1, CH	1.46 ^a^, m	50	17.3, CH_3_	1.23, d (7.0)
20	23.2, CH_3_	0.86, d (6.5)	(*S*,*S*)-EPCA		
21	21.8, CH_3_	0.85, d (6.5)	51	169.2, C	
l-Pro			52	124.6, CH	6.94, d (15.5)
22	171.8, C		53	136.69, CH	8.11, d (15.5)
23	60.2, CH	4.37, dd (7.5, 7.0)	54	134.1, C	
24a	30.0, CH_2_	2.21, m	55	126.9, CH	7.42 ^a^, d (7.5)
24b		1.56, m	56	127.8, CH	7.00, ddd (7.5, 7.5, 1.0)
25	24.7, CH_2_	1.79, m	57	129.6, CH	7.33, ddd (7.5, 7.5, 1.0)
26a	47.3, CH_2_	3.72, m	58	124.4, CH	7.09 ^a^, dd (7.5, 1.0)
26b		3.52, m	59	136.74, C	
(*S*,*S*)-Hle			60	54.8, CH	4.07, d (2.0)
27	170.1, C		61	58.9, CH	2.97, qd (5.0, 2.0)
28	52.5, CH	4.59, dd (9.5, 9.5)	62	17.6, CH_3_	1.37, d (5.0)

^a^ Overlapped signals.

## Data Availability

All data is contained within this article and Supplementary Materials.

## Supplementary Materials

The following supporting information can be downloaded at: https://www.mdpi.com/article/10.3390/md20070455/s1: Figure S1. Biosynthetic mechanisms of (a) nonribosomal peptide synthetase (NRPS) and (b) polyketide synthetase (PKS); Figure S2. Structures of CCNPs from actinomycetes; Figure S3. HRESIMS data of the epoxinnamide (**1**); Figure S4. ^1^H NMR (800 MHz, DMSO-*d*_6_) spectrum of the epoxinnamide (**1**); Figure S5. ^13^C NMR (200 MHz, DMSO-*d*_6_) spectrum of the epoxinnamide (**1**); Figure S6. HSQC NMR (800 MHz, DMSO-*d*6) spectrum of the epoxinnamide (**1**); Figure S7. Structure comparison between epoxinnamide (**1**) and nyuzenamide C; Figure S8. COSY NMR (800 MHz, DMSO-*d*_6_) spectrum of the epoxinnamide (**1**); Figure S9. TOCSY NMR (800 MHz, DMSO-*d*_6_) spectrum of the epoxinnamide (**1**); Figure S10. HMBC NMR (800 MHz, DMSO-*d*_6_) spectrum of the epoxinnamide (**1**); Figure S11. ROESY NMR (800 MHz, DMSO-*d*_6_) spectrum of the epoxinnamide (**1**); Figure S12. 16S rRNA gene sequence-based neighbor-joining tree showing the phylogenetic position of the strain OID44; Figure S13. LC/MS chromatograms of l- and d-FDAA derivatives of amino acids in the epoxinnamide (**1**); Figure S14. LC/MS chromatograms of d-FDAA derivatives of threonine in the epoxinnamide (**1**) and authentic threonines; Figure S15. ^1^H, ^13^C, and ^3^*J*_H2-H3_ values of hydroxyleucine moiety in various natural products; Figure S16. Results of DP4 calculation for the partial structure of the epoxinnamide (**1**); Figure S17. Biosynthetic functions of the cytochrome P450s and the thioesterase domains in epoxinnamide (**1**), nyuzenamide C, skyllamycin A, atratumycin, and telomycin; Figure S18. Sequence alignment of the Epc-TE with the other TEs; Figure S19. Proposed structural models of thioesterase domains, active site residues surrounding catalytic Ser residue, and key active sites for dual function of (a) Epc-TE, (b) Dml-TE, (c) Atr-TE, and (d) Tel-TE; Figure S20. Sequence alignment of the EpcB with the other cytochrome P450s; Figure S21. Phylogenetic analysis of the cytochrome P450 *β*-hydroxylases which catalyze *β*-hydroxylation of amino acid; Figure S22. Phylogenetic tree and table of the ketosynthases in BGC of youssoufene and CCNPs; Table S1. ^1^H (800 MHz, DMSO-*d*_6_) and ^13^C NMR (200 MHz, DMSO-*d*_6_) comparison table between epoxinnamide (**1**) and nyuzenamide C; Table S2. LC/MS analysis of l- and d-FDAA derivatives of amino acids in the epoxinnamide (**1**); Table S3. Experimental and calculated chemical shifts of the partial structure of the epoxinnamide (**1**); Table S4. AntiSMASH output table of the *Streptomyces* sp. OID44; Table S5. Deduced functions of ORFs in the epoxinnamide (**1**) biosynthetic gene cluster from the *Streptomyces* sp. OID44; Table S6. List of the cytochrome P450 *β*-hydroxylases implicated in the *β*-hydroxylation of amino acid residues; Table S7. Quinone reductase assay data of nyuzenamide C and epoxinnamide (**1**); Table S8. Cell viability data of nyuzenamide C and epoxinnamide (**1**); Table S9. In vitro capillary tube formation assay data of nyuzenamide C and epoxinnamide (**1**).
