# Supplementary material for: Epoxinnamide: An Epoxy Cinnamoyl-Containing Nonribosomal Peptide from an Intertidal Mudflat-Derived Streptomyces sp
Source: Mar Drugs. 2022 Jul 12;20(7):455. doi: 10.3390/md20070455 (PMC9321520; doi:10.3390/md20070455)
Supplement: Supplementary file 1 [file marinedrugs-20-00455-s001.zip › marinedrugs-1752412-supplementary.pdf]

Supplementary Materials

Epoxinnamide: An Epoxy Cinnamoyl-Containing Nonribosomal Peptide from an Intertidal Mud-flat-Derived *Streptomyces* sp.

Sangwook Kang [1], Jaeho Han [1], Sung Chul Jang [1], Joon Soo An [1], Ilnam Kang [2], Yun Kwon [3], Sang-Jip Nam [4], Sang Hee Shim [1], Jang-Cheon Cho [2], Sang Kook Lee [1] and Dong-Chan Oh [1,*]

[1] Natural Products Research Institute, College of Pharmacy, Seoul National University, Seoul 08826, Korea; ksw1657@snu.ac.kr (S.K.); gh03292@snu.ac.kr (J.H.); tobok95@snu.ac.kr (S.C.J.); ahnjun-soo@snu.ac.kr (J.S.A.); sanghee_shim@snu.ac.kr (S.H.S); sklee61@snu.ac.kr (S.K.L)
[2] Department of Biological Sciences, Inha University, Incheon 22212, Korea; ikang@inha.ac.kr (I.K.); chojc@inha.ac.kr (J.-C.C.)
[3] Research Institute of Pharmaceutical Sciences, College of Pharmacy, Kyungpook National University, Daegu 41566, Korea; yunkwon@knu.ac.kr
[4] Department of Chemistry and Nanoscience, Ewha Womans University, Seoul 03760, Korea; sjnam@ewha.ac.kr
* Correspondence: dongchanoh@snu.ac.kr; Tel.: +82-880-2491; Fax: +82-762-8322

Contents

Figure S1. Biosynthetic mechanisms of (**a**) nonribosomal peptide synthetase (NRPS) and (**b**) polyketide synthetase (PKS). S4

Figure S2. Structures of CCNPs from actinomycetes. S5

Figure S3. HRESIMS data of the epoxinnamide (**1**). S6

Figure S4. ^{1}H NMR (800 MHz, DMSO-d_6) spectrum of the epoxinnamide (**1**). S7

Figure S5. ^{13}C NMR (200 MHz, DMSO-d_6) spectrum of the epoxinnamide (**1**). S8

Figure S6. HSQC NMR (800 MHz, DMSO-d_6) spectrum of the epoxinnamide (1). S9

Figure S7. Structure comparison between epoxinnamide (**1**) and nyuzenamide C. S10

Table S1. ^{1}H (800 MHz, DMSO-d_6) and ^{13}C NMR (200 MHz, DMSO-d_6) comparison table between epoxinnamide (**1**) and nyuzenamide C. S10

Figure S8. COSY NMR (800 MHz, DMSO-d_6) spectrum of the epoxinnamide (**1**). S13

Figure S9. TOCSY NMR (800 MHz, DMSO-d_6) spectrum of the epoxinnamide (**1**). S14

Figure S10. HMBC NMR (800 MHz, DMSO-d_6) spectrum of the epoxinnamide (**1**). S15

Figure S11. ROESY NMR (800 MHz, DMSO-d_6) spectrum of the epoxinnamide (**1**). S16

Figure S12. 16S rRNA gene sequence-based neighbor-joining tree showing the phylogenetic position of the strain OID44. .... S17

Figure S13. LC/MS chromatograms of L- and D-FDAA derivatives of amino acids in the epoxinnamide (**1**). S18

Table S2. LC/MS analysis of L- and D-FDAA derivatives of amino acids in the epoxinnamide (**1**). S19

Figure S14. LC/MS chromatograms of D-FDAA derivatives of threonine in the epoxinnamide (**1**) and authentic threonines. S19

Figure S15. ^{1}H, ^{13}C, and $^{3}J_{H2\text{-}H3}$ values of hydroxyleucine moiety in various natural products. S20

Table S3. Experimental and calculated chemical shifts of the partial structure of the epoxinnamide (**1**). S23

Figure S16. Results of DP4 calculation for the partial structure of the epoxinnamide (**1**). S24

Table S4. AntiSMASH output table of the *Streptomyces* sp. OID44. .... S25

Table S5. Deduced functions of ORFs in the epoxinnamide (**1**) biosynthetic gene cluster from the *Streptomyces* sp. OID44. .... S27

Figure S17. Biosynthetic functions of the cytochrome P450s and the thioesterase domains in epoxinnamide (**1**), nyuzenamide C, skyllamycin A, atratumycin, and telomycin. S29

Figure S18. Sequence alignment of the Epc-TE with the other TEs..S30

Figure S19. Proposed structural models of thioesterase domains, active site residues surrounding catalytic Ser residue, and key active sites for dual function of (**a**) Epc-TE, (**b**) Dml-TE, (**c**) Atr-TE, and (**d**) Tel-TE...S31

Figure S20. Sequence alignment of the EpcB with the other cytochrome P450s. S32

Figure S21. Phylogenetic analysis of the cytochrome P450 *β*-hydroxylases which catalyze *β*-hydroxylation of amino acid. S33

Table S6. List of the cytochrome P450 *β*-hydroxylases implicated in the *β*-hydroxylation of amino acid residues. S34

Figure S22. Phylogenetic tree and table of the ketosynthases in BGC of youssoufene and CCNPs....S35

Table S7. Quinone reductase assay data of nyuzenamide C and epoxinnamide (**1**). S36

Table S8. Cell viability data of nyuzenamide C and epoxinnamide (**1**). S36

Table S9. In vitro capillary tube formation assay data of nyuzenamide C and epoxinnamide (**1**)......S36

References S37

Figure S1. Biosynthetic mechanisms of (**a**) nonribosomal peptide synthetase (NRPS) and (**b**) polyketide synthetase (PKS) [1]. Wavy bonds at the PCP and ACP domains represent the 4′-phosphopantetheine groups. ATP = adenosine triphosphate, PP_i = pyrophosphate, AMP = adenosine monophosphate, CoA = coenzyme A, R_1, R_2, R_3 = amino acid residues, R = poly-β-keto group.

(a)

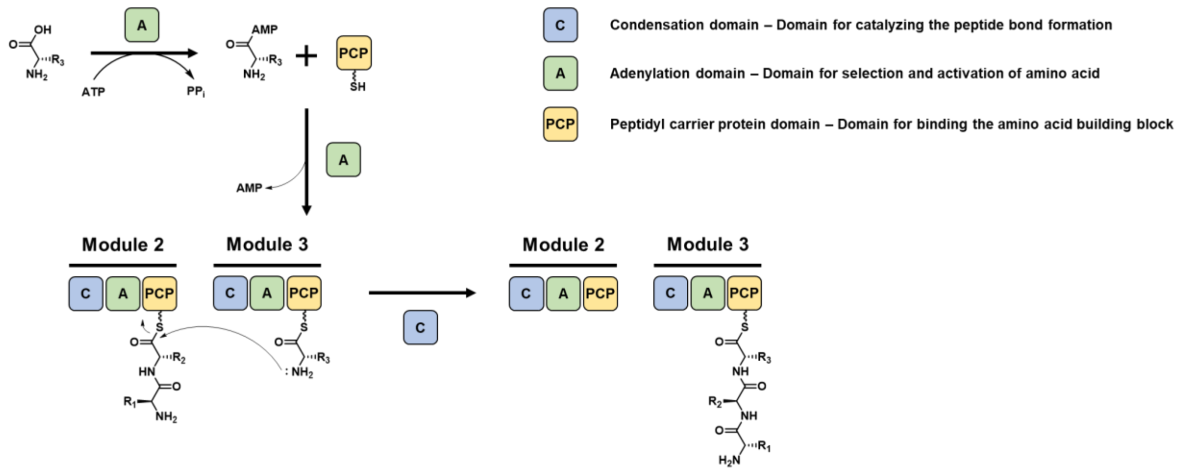

(b)

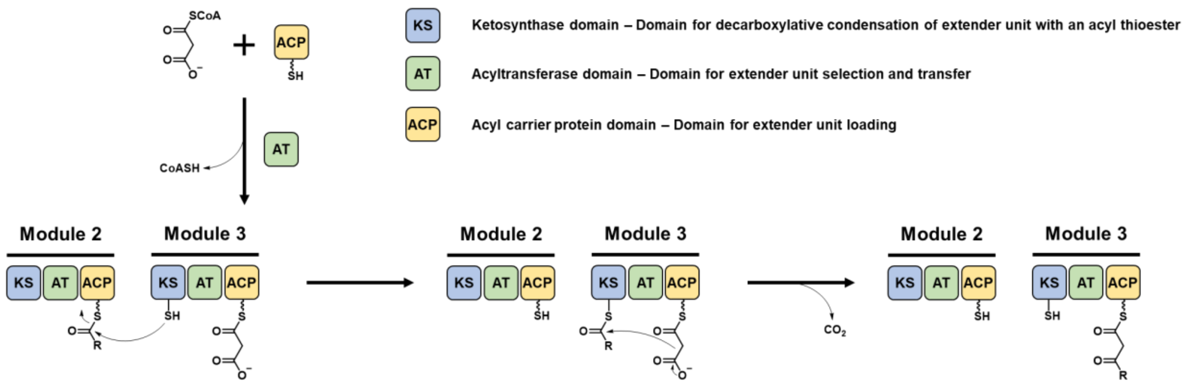

Figure S2. Structures of CCNPs from actinomycetes [2-21]. Cinnamoyl moieties are highlighted by red color.

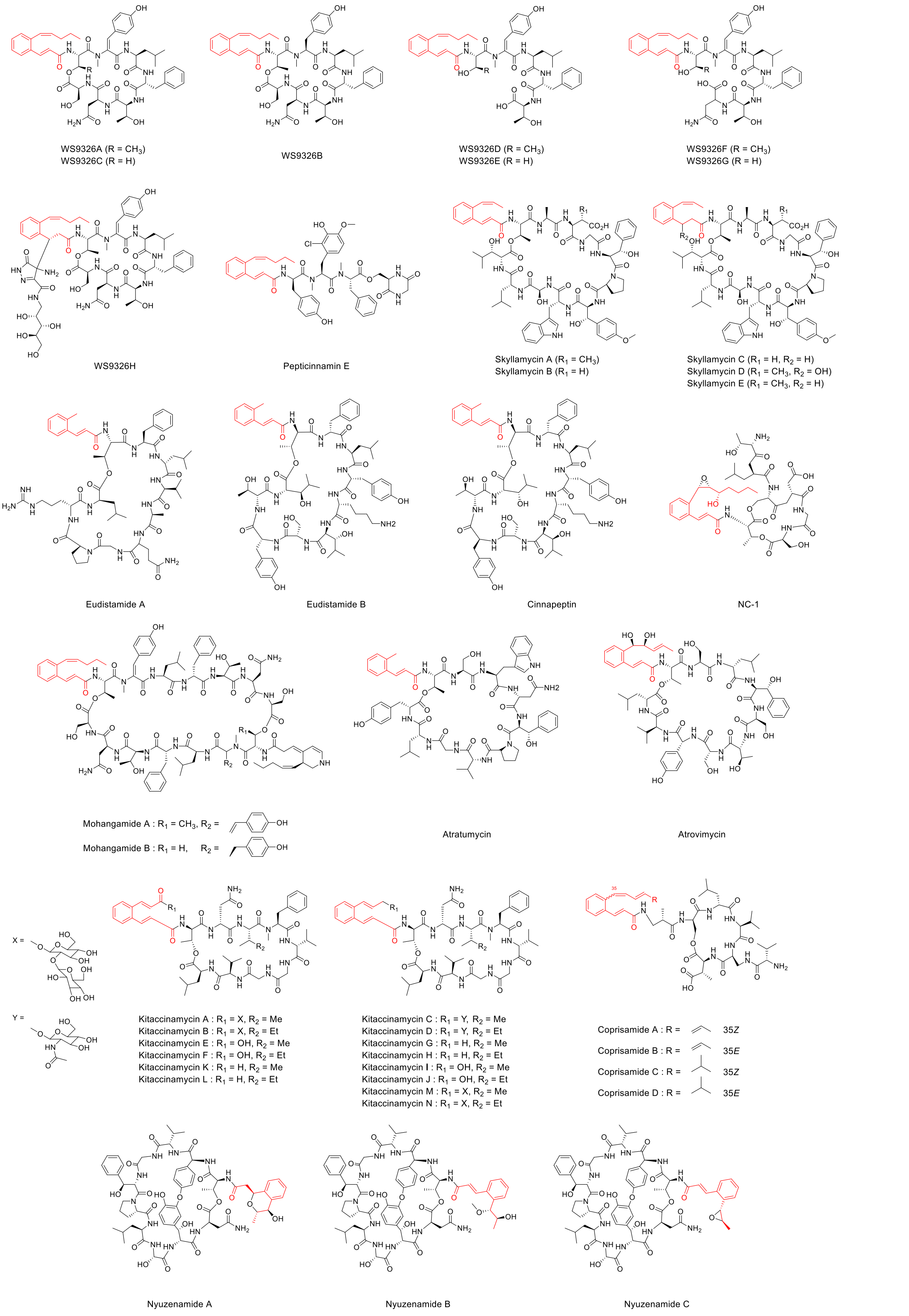

Figure S3. HRESIMS data of the epoxinnamide (**1**).

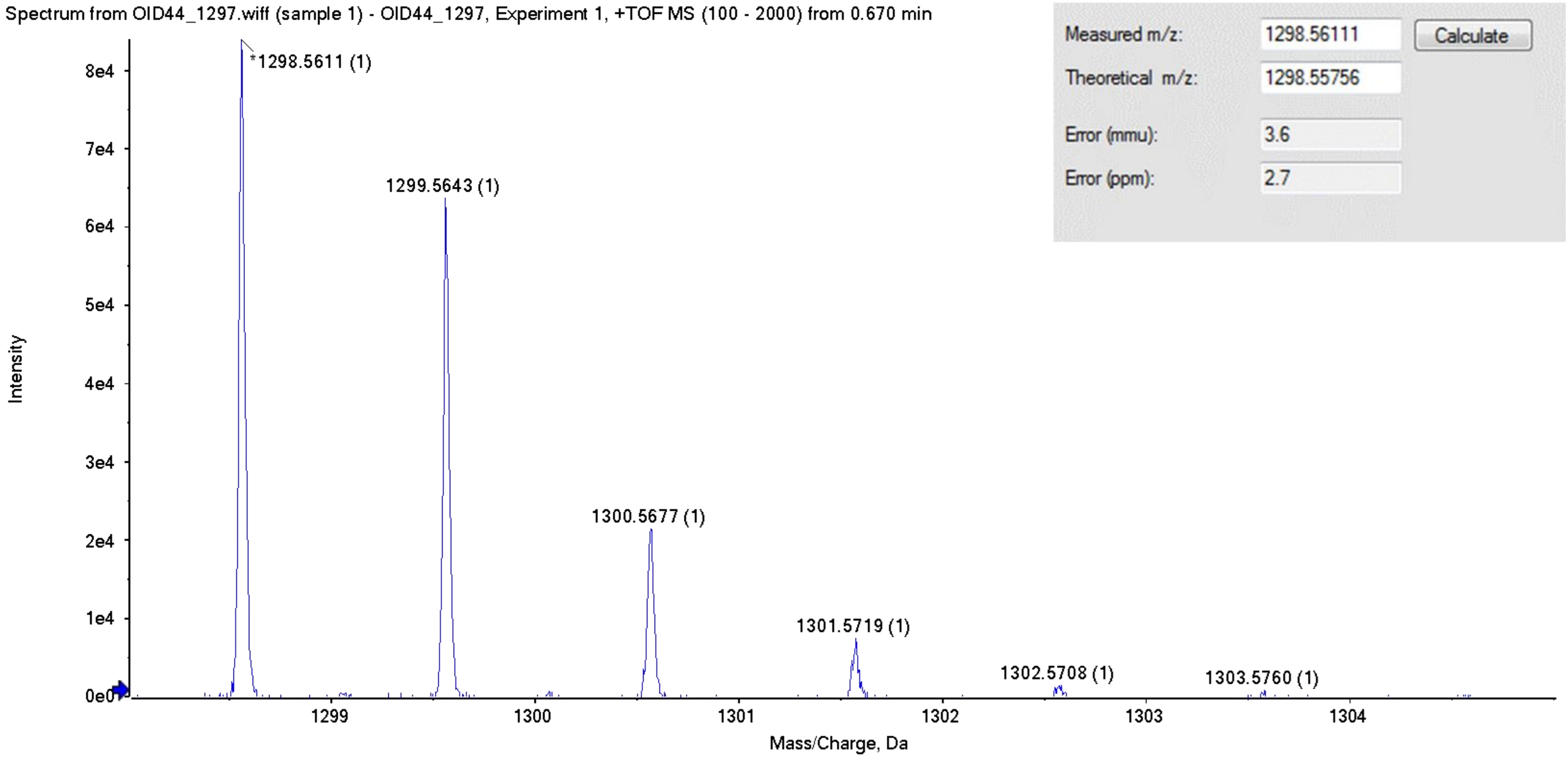

Figure S4. ^{1}H NMR (800 MHz, DMSO-d_6) spectrum of the epoxinnamide (**1**).

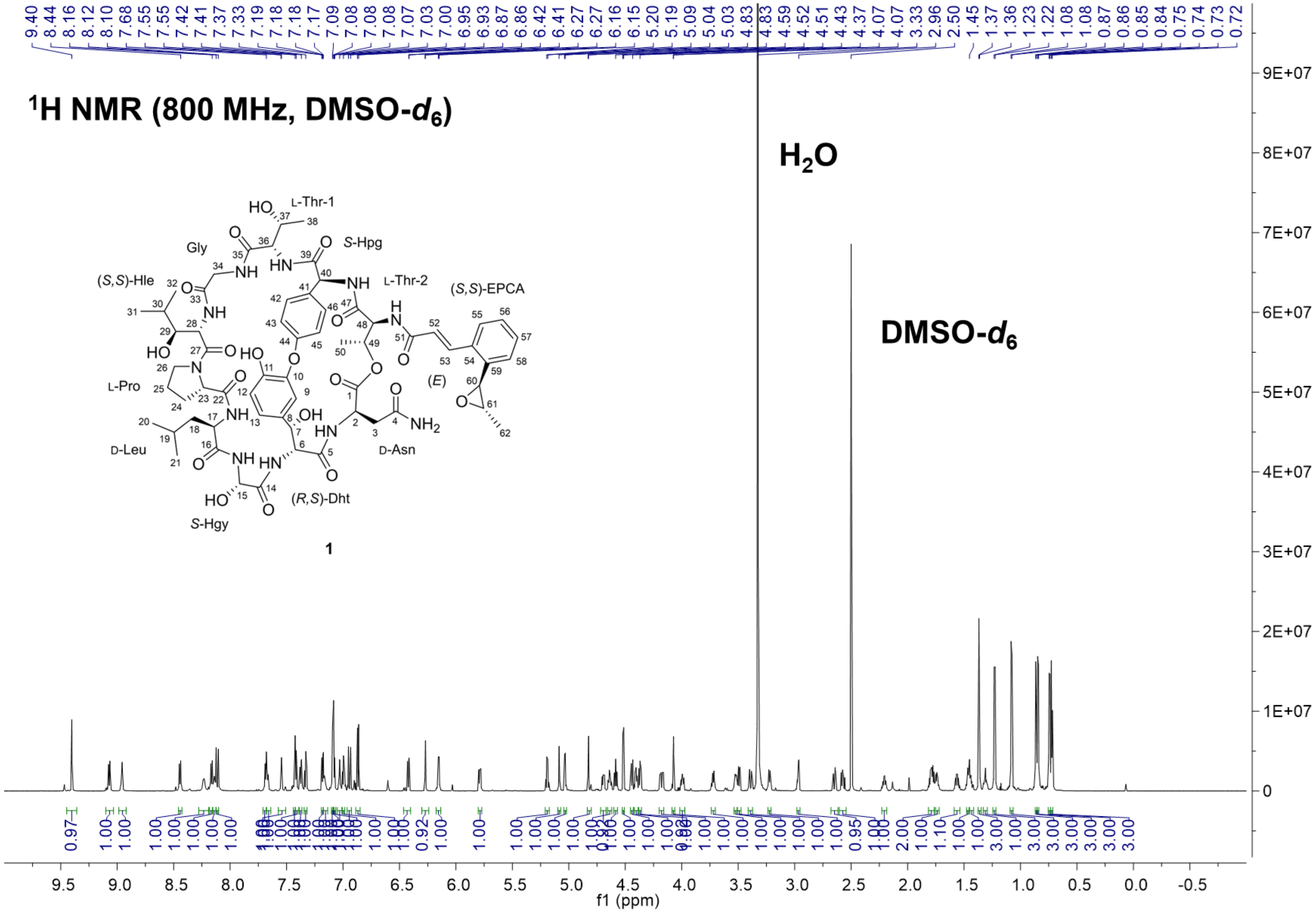

Figure S5. ^{13}C NMR (200 MHz, DMSO-d_6) spectrum of the epoxinnamide (**1**).

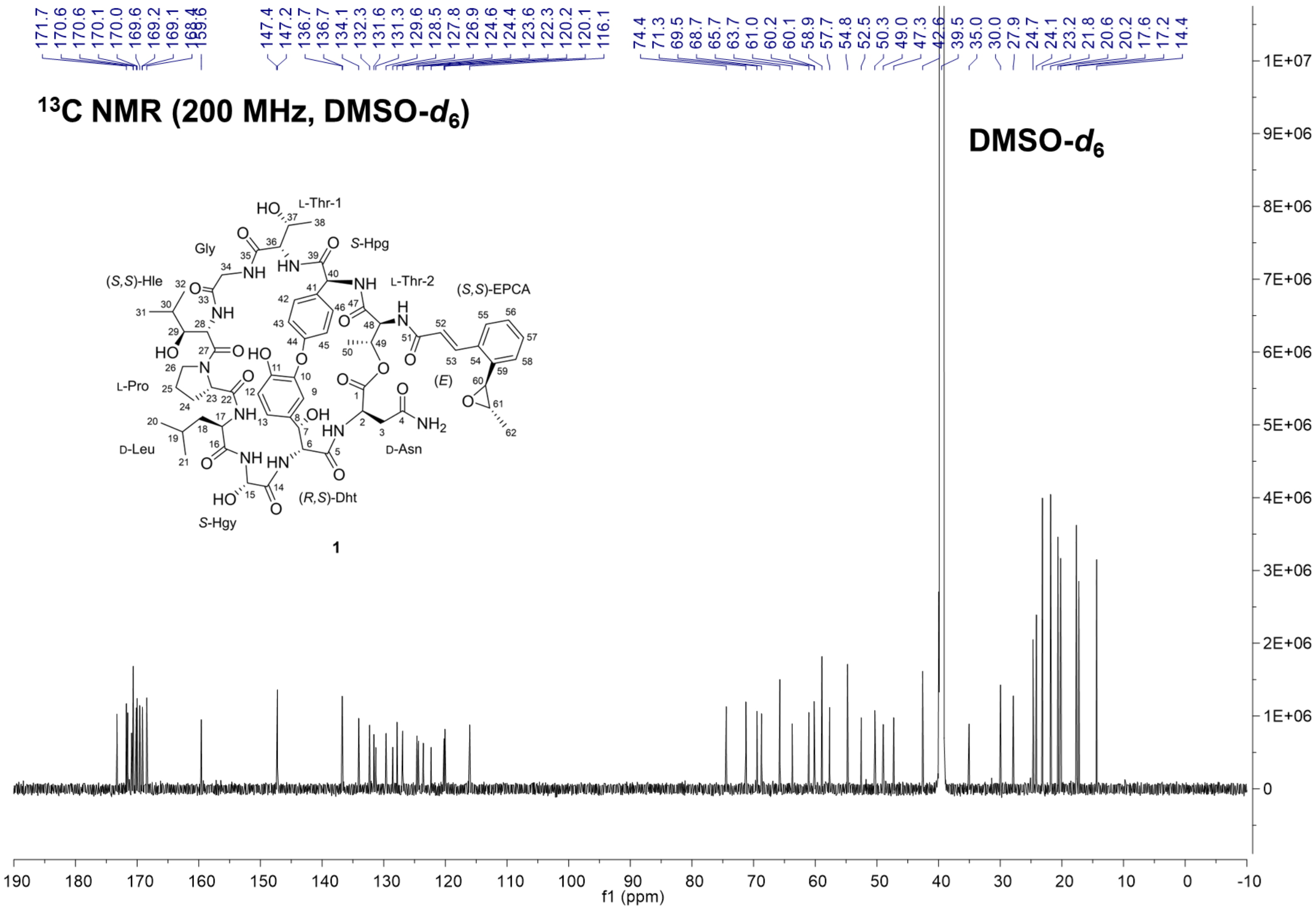

Figure S6. HSQC NMR (800 MHz, DMSO-d_6) spectrum of the epoxinnamide (1).

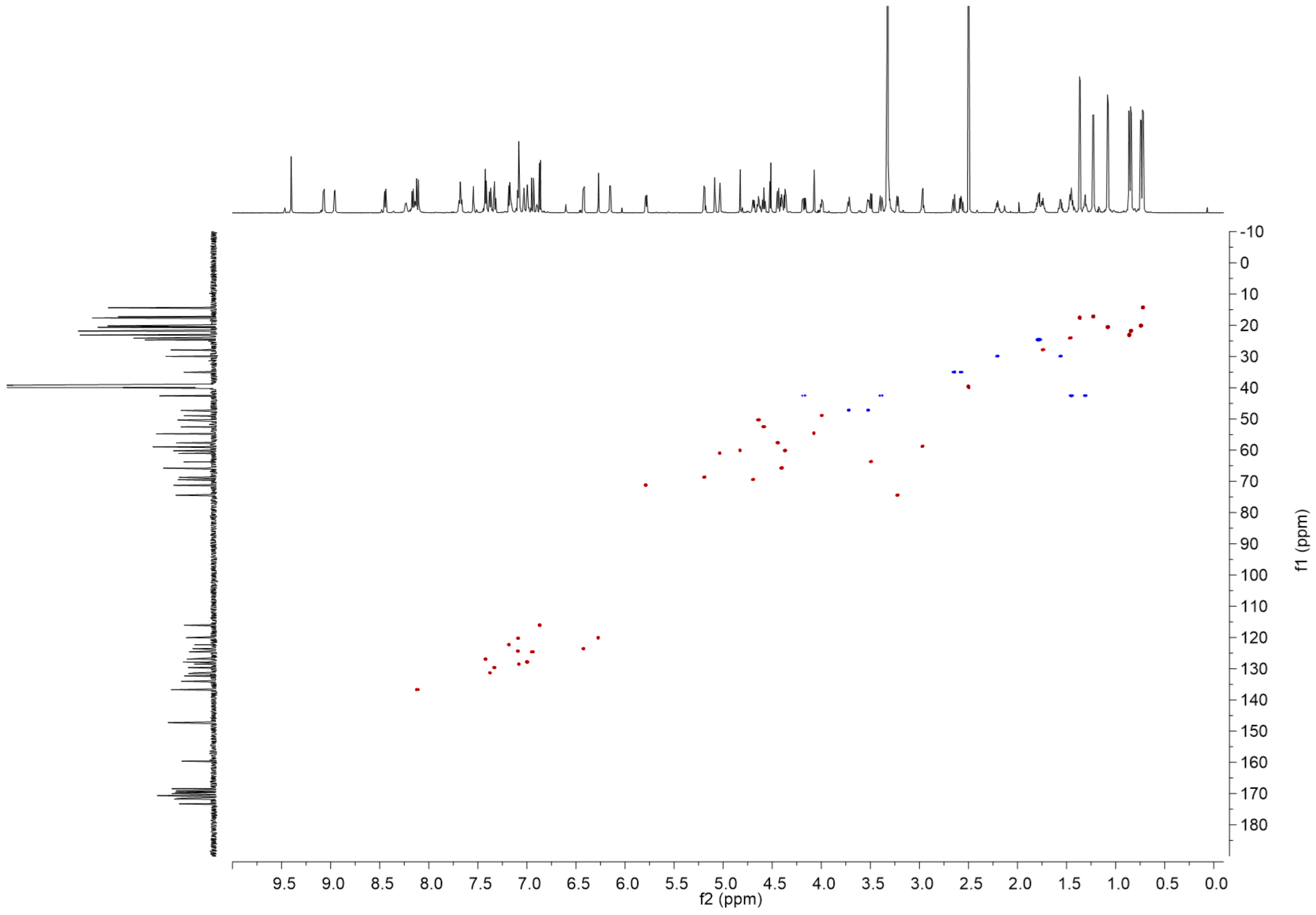

Figure S7. Structure comparison between epoxinnamide (**1**) and nyuzenamide C [21].

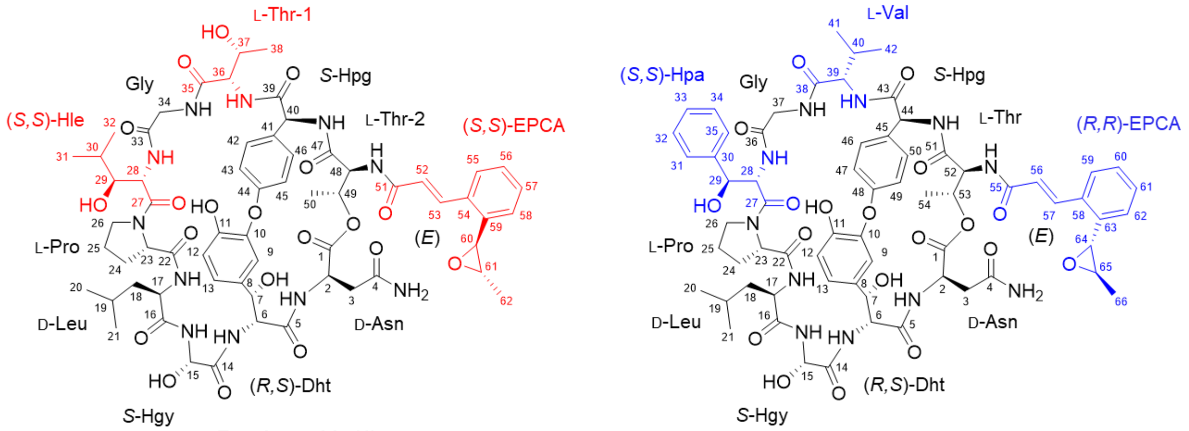

Epoxinnamide (1) **Nyuzenamide C**

Table S1. ^{1}H (800 MHz, DMSO-d_6) and ^{13}C NMR (200 MHz, DMSO-d_6) comparison table between epoxinnamide (**1**) and nyuzenamide C [21].

**Epoxinnamide (1)**			**Nyuzenamide C**			**Δδ$_C$**	**Δδ$_H$**
**position**	**δ$_{C,E}$**	**δ$_{H,E}$**	**position**	**δ$_{C,N}$**	**δ$_{H,N}$**	**(= δ$_{C,E}$ - δ$_{C,N}$)**	**(=δ$_{H,E}$ – δ$_{H,N}$)**
D-Asn			D-Asn				
1	169.6		1	169.6		0	
2	49.0	3.99	2	49.1	3.95	-0.1	0.04
2-NH		8.23	2-NH		8.27		-0.04
3a	35.1	2.65	3a	34.9	2.70	0.2	-0.05
3b		2.57	3b		2.61		-0.04
4	170.64		4	170.7		-0.1	
4-NH$_2$a		7.43	4-NH$_2$a		7.47		-0.04
4-NH$_2$b		7.03	4-NH$_2$b		7.06		-0.03
(*R*,*S*)-Dht			(*R*,*S*)-Dht				
5	170.63		5	170.7		-0.1	
6	63.8	3.49	6	64.2	3.54	-0.4	-0.05
6-NH		8.14	6-NH		8.16		-0.02
7	69.5	4.69	7	69.6	4.72	-0.1	-0.03
7-OH		6.15	7-OH		6.24		-0.09
8	132.3		8	132.7		-0.4	
9	120.1	6.27	9	120.8	6.34	-0.7	-0.07
10	147.3		10	147.2		0.1	
11	147.4		11	147.7		-0.3	
11-OH		9.40	11-OH		9.46		-0.06
12	116.1	6.87	12	116.2	6.97	-0.1	-0.10
13	120.2	7.08	13	120.1	7.21	0.1	-0.13
S-Hgy			*S*-Hgy				
14	170.9		14	171.0		-0.1	
15	71.3	5.79	15	71.1	5.77	0.2	0.02

15-OH		7.17	15-OH		7.13		0.04
15-NH		9.07	15-NH		9.04		0.03
D-Leu			D-Leu				
16	171.5		16	171.4		0.1	
17	50.3	4.64	17	50.3	4.58	0	0.06
17-NH		8.44	17-NH		8.53		-0.09
18a	42.58	1.45	18a	42.3	1.44	0.3	0.01
18b		1.31	18b		1.28		0.03
19	24.1	1.46	19	24.1	1.45	0	0.01
20	23.2	0.86	20	23.1	0.85	0.1	0.01
21	21.8	0.85	21	21.8	0.81	0	0.04
L-Pro			L-Pro				
22	171.8		22	171.5		0.3	
23	60.2	4.37	23	60.4	4.40	-0.2	-0.03
24a	30.0	2.21	24a	29.8	2.26	0.2	-0.05
24b		1.56	24b		1.63		-0.07
25a	24.7	1.79	25a	24.8	1.89	-0.1	-0.10
25b		1.79	25b		1.82		-0.03
26a	47.3	3.72	26a	47.5	3.92	-0.2	-0.20
26b		3.52	26b		3.66		-0.14
(*S*,*S*)-Hle			(*S*,*S*)-Hpa				
27	170.1		27	169.1			
28	52.5	4.59	28	57.5	4.53		
28-NH		7.69	28-NH		7.90		
29	74.4	3.22	29	73.3	4.32		
29-OH		5.09	29-OH		5.75		
30	27.9	1.74	30	141.7			
31	14.4	0.72	31	126.4	7.48		
32	20.2	0.74	32	127.8	7.27		
			33	127.5	7.27		
			34	127.8	7.27		
			35	126.4	7.48		
Gly			Gly				
33	168.4		36	168.7		-0.3	
34a	42.60	4.18	37a	42.8	3.98	-0.2	0.20
34b		3.39	37b		3.30		0.09
34-NH		7.68	37-NH		7.50		0.18
L-Thr-1			L-Val				
35	170.0		38	170.4			
36	57.7	4.44	39	56.7	4.56		
36-NH		8.16	39-NH		7.84		
37	65.8	4.40	40	28.9	2.65		
37-OH		4.52	41	17.2	1.16		
38	20.3	1.08	42	19.8	0.80		

S-Hpg			*S*-Hpg				
39	169.1		43	169.2		-0.1	
40	60.1	4.83	44	60.3	4.77	-0.2	0.06
40-NH		7.55	44-NH		7.67		-0.12
41	131.6		45	131.5		0.1	
42	128.5	7.07	46	127.9	7.05	0.6	0.02
43	123.6	6.42	47	123.5	6.51	0.1	-0.09
44	159.6		48	160.0		-0.4	
45	122.3	7.18	49	122.3	7.25	0	-0.07
46	131.3	7.37	50	131.1	7.44	0.2	-0.07
L-Thr-2			L-Thr				
47	173.3		51	173.0		0.3	
48	61.0	5.03	52	60.7	4.96	0.3	0.07
48-NH		8.96	52-NH		9.00		-0.04
49	68.7	5.19	53	69.0	5.22	-0.3	-0.03
50	17.3	1.23	54	17.5	1.27	-0.2	-0.04
(*S*,*S*)-EPCA			(*R*,*R*)-EPCA				
51	169.2		55	168.4		0.8	
52	124.6	6.94	56[a]	127.2[a]	6.98[a]	-2.6	-0.04
53	136.7	8.11	57[a]	135.8[a]	7.91[a]	0.9	0.20
54	134.1		58	134.9		-0.8	
55	126.9	7.42	59	126.9	7.04	0	0.38
56	127.8	7.00	60	128.3	7.09	-0.5	-0.09
57	129.6	7.33	61	129.7	7.36	-0.1	-0.03
58	124.4	7.09	62	124.7	7.07	-0.3	0.02
59	136.7		63	135.9		0.8	
60	54.8	4.07	64	54.8	3.31	0	0.76
61	58.9	2.97	65	57.8	2.74	1.1	0.23
62	17.6	1.37	66	16.3	0.54	1.3	0.83

[a] Previously-reported data [21] would have to be exchanged.

Figure S8. COSY NMR (800 MHz, DMSO-d_6) spectrum of the epoxinnamide (**1**).

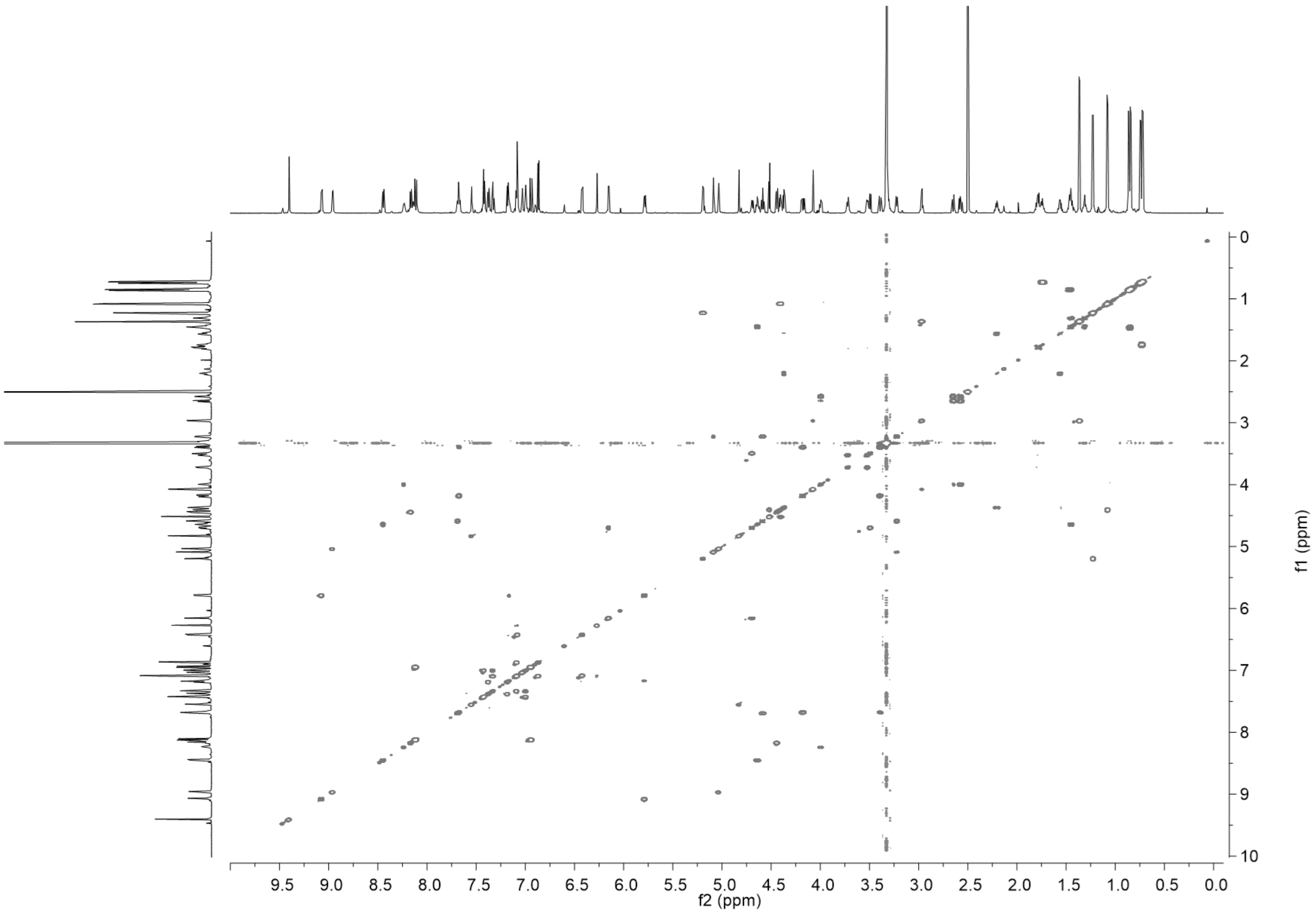

Figure S9. TOCSY NMR (800 MHz, DMSO-d_6) spectrum of the epoxinnamide (**1**).

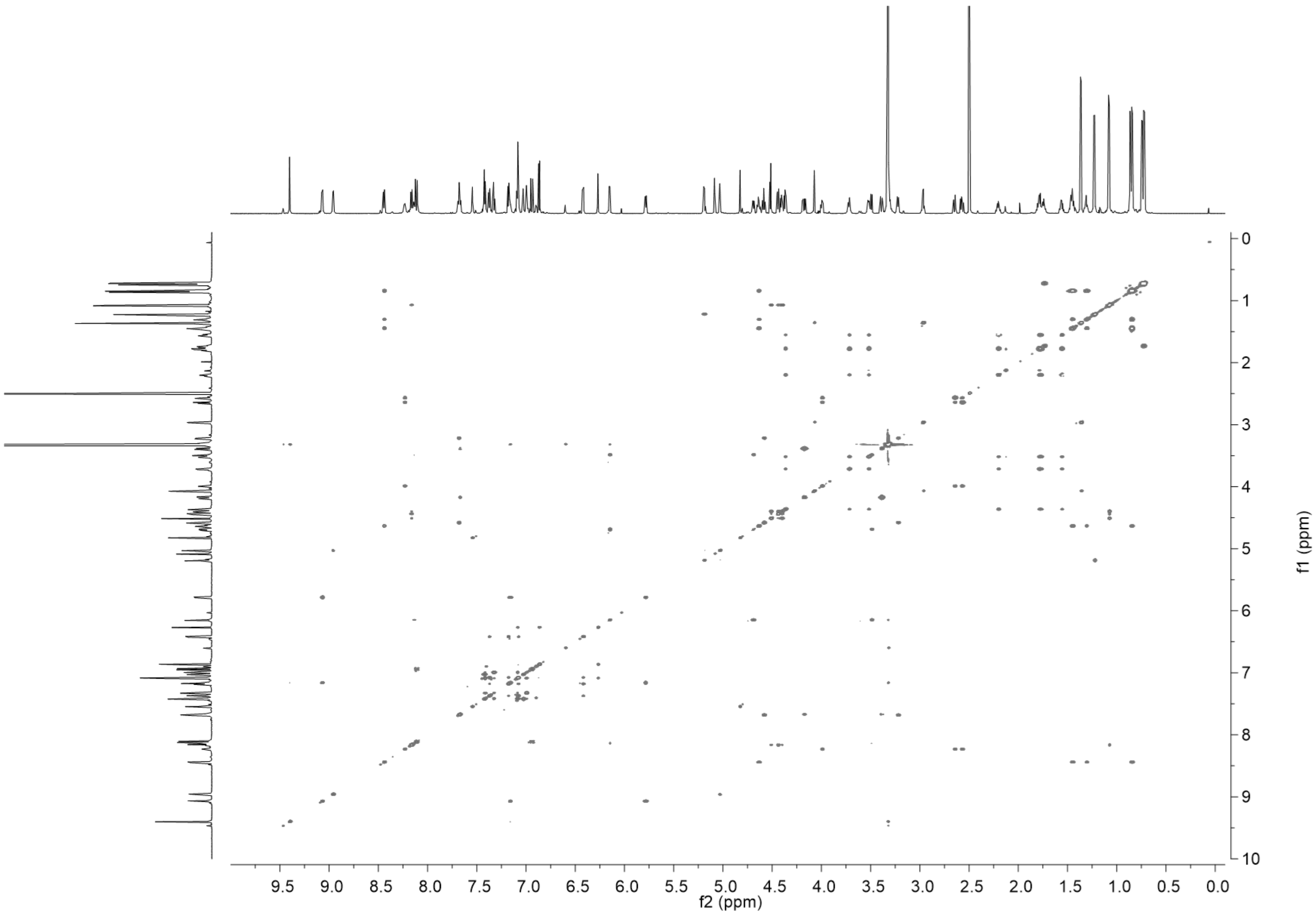

Figure S10. HMBC NMR (800 MHz, DMSO-d_6) spectrum of the epoxinnamide (**1**).

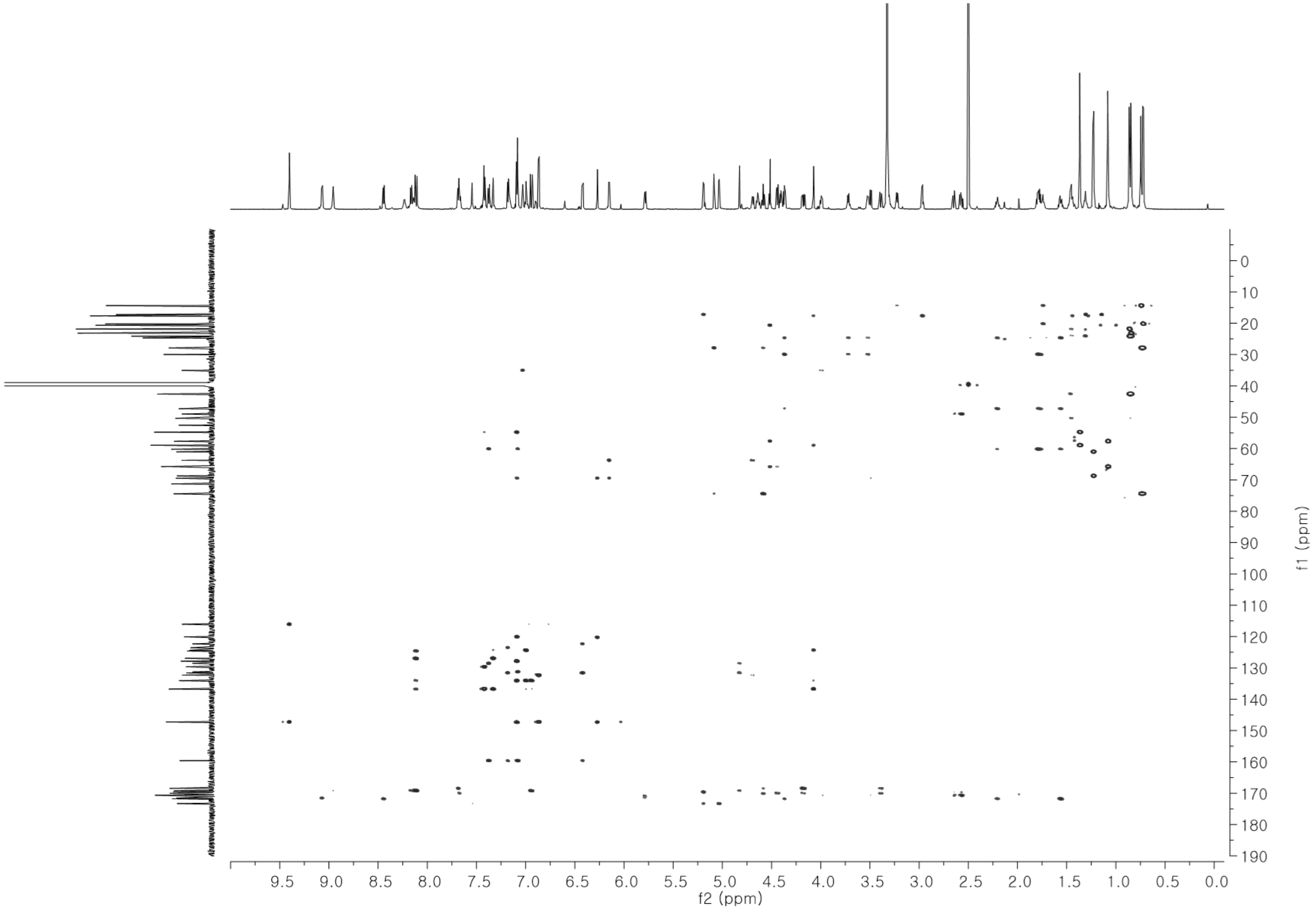

Figure S11. ROESY NMR (800 MHz, DMSO-d_6) spectrum of the epoxinnamide (**1**).

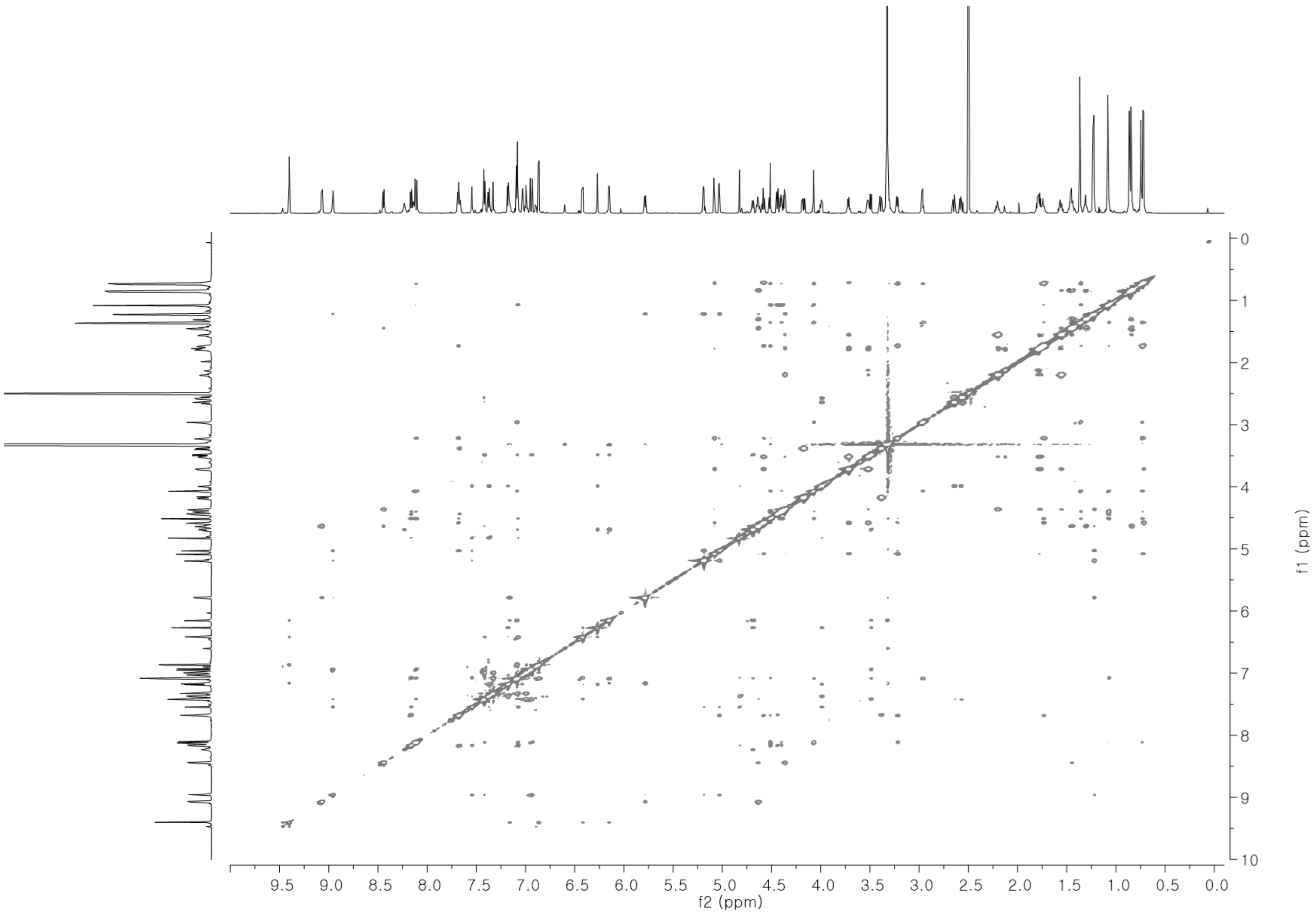

Figure S12. 16S rRNA gene sequence-based neighbor-joining tree showing the phylogenetic position of the strain OID44 (marked in red and bold). Nyuzenamide-producing strains N11-34 and DM14 are marked in bold. The evolutionary distances were computed using the maximum composite likelihood method with the rate variation among sites modeled with a gamma distribution. Bootstrap supporting values (≥50%; 100 replicates) are shown on the branches. The scale bar indicates the average number of base substitutions per site.

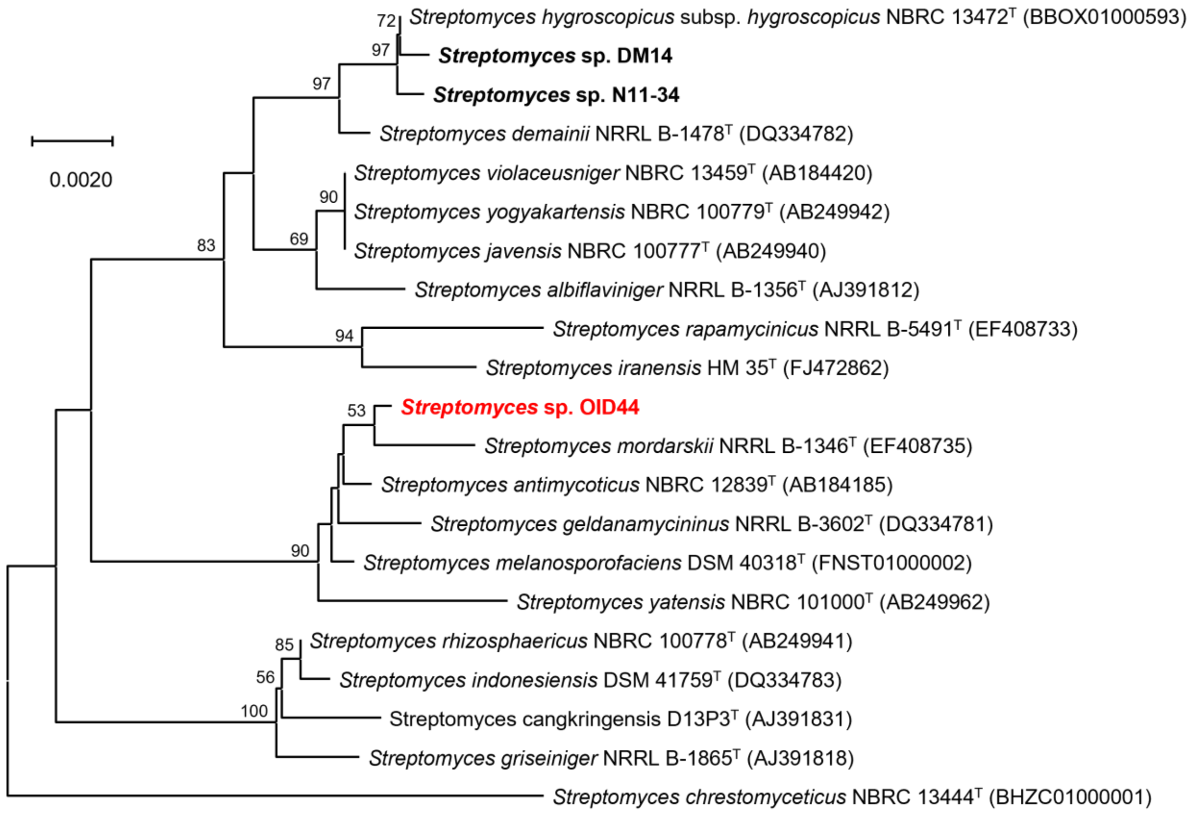

Figure S13. LC/MS chromatograms of L- and D-FDAA derivatives of amino acids in the epoxinnamide (**1**). (**a**) asparagine (aspartic acid), (**b**) leucine, (**c**) proline, (**d**) *β*-hydroxyleucine, (**e**) threonine

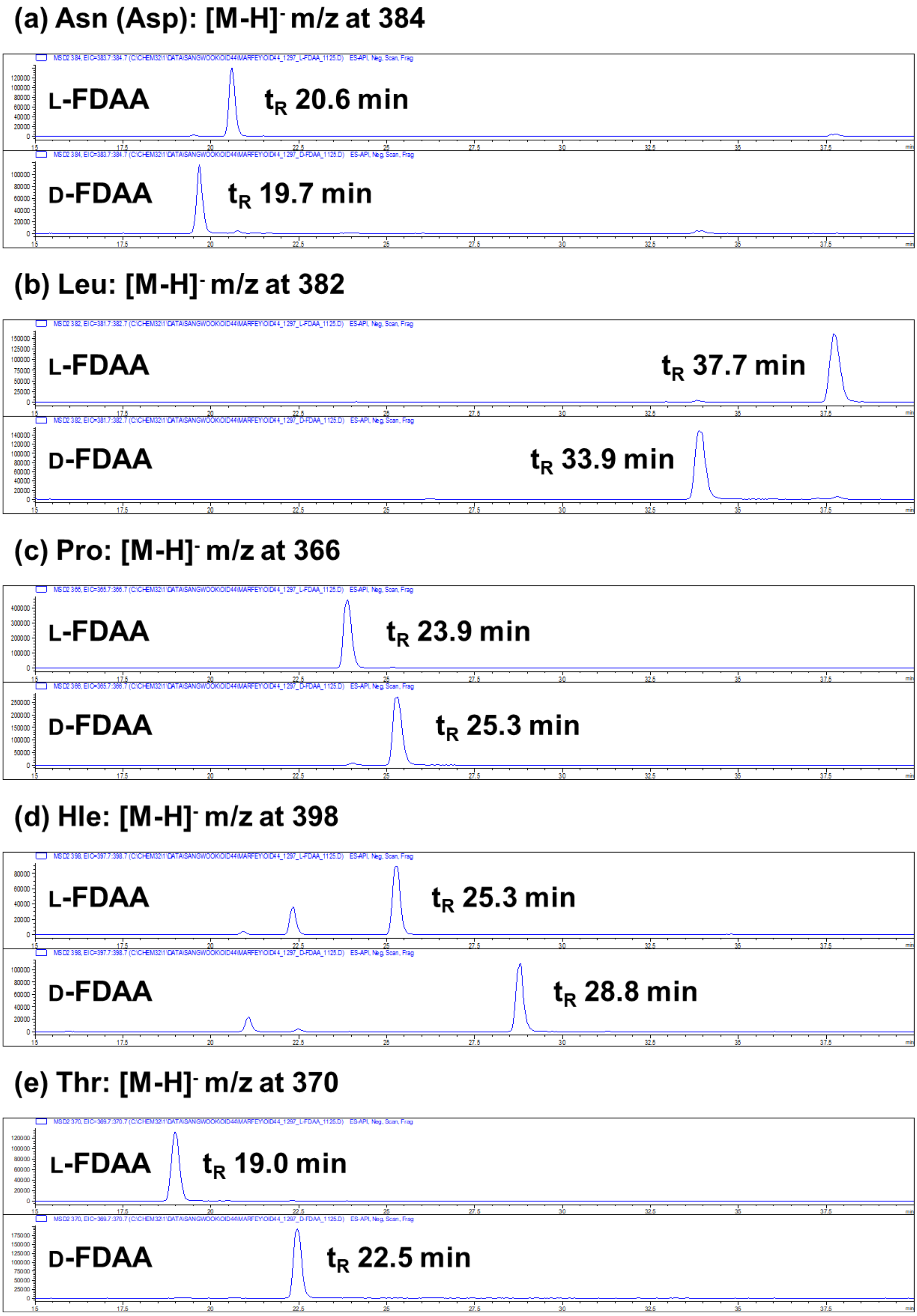

Table S2. LC/MS analysis of L- and D-FDAA derivatives of amino acids in the epoxinnamide (**1**).

Amino Acids	t_{RL} (min)	t_{RD} (min)	Elution Order	Δt (=t_{RD}-t_{RL}, min)
Asn (Asp)	20.6	19.7	D → L	-0.9
Leu	37.7	33.9	D → L	-3.8
Pro	23.9	25.3	L → D	1.4
Hle	25.3	28.8	L → D	3.5
Thr	19.0	22.5	L → D	3.5

Figure S14. LC/MS chromatograms of D-FDAA derivatives of threonine in the epoxinnamide (**1**) and authentic threonines. (**a**) D-FDAA derivative of threonine in the epoxinnamide, (**b**) D-FDAA derivative of authentic L-Threonine, (**c**) D-FDAA derivative of authentic L-*allo*-Threonine

(a) D-FDAA derivative of threonine in the epoxinnamide (1)

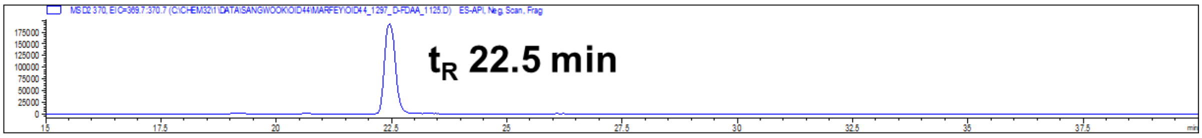

(b) D-FDAA derivative of authentic L-Thr

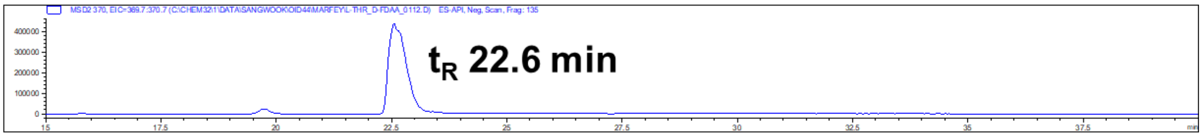

(c) D-FDAA derivative of authentic L-*allo*-Thr

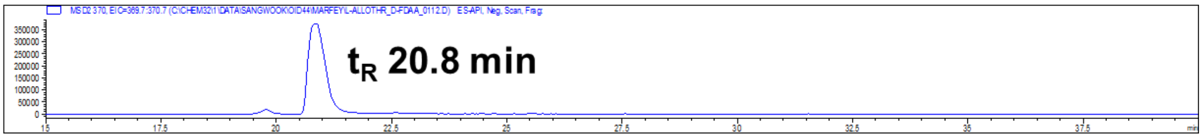

Figure S15. ^{1}H, ^{13}C, and $^{3}J_{H2\text{-}H3}$ values of hydroxyleucine moiety in various natural products. (**a**) (2*S*,3*S*)-hydroxyleucine, (**b**) (2*R*,3*R*)-hydroxyleucine, (**c**) (2*S*,3*R*)-hydroxyleucine, (**d**) (2*R*,3*S*)-hydroxyleucine [22-32].

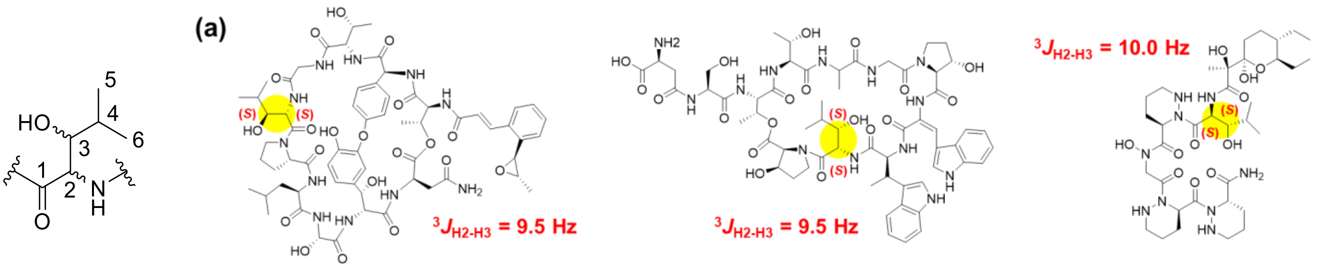

Epoxinnamide (**1**)   Telomycin   Dentigerumycin D

Compounds	Epoxinnamide (1)[a]		Telomycin[b]		Dentigerumycin D[c]	
Position	δC	δH (J in Hz)	δC	δH (J in Hz)	δC	δH (J in Hz)
1	170.1				172.4	
2	52.5	4.59, dd (9.5, 9.5)	51.6	4.54, (9.5)	49.6	5.48, dd (10.0, 1.0)
2-NH		7.69, d (9.5)				7.66, d (10.0)
3	74.4	3.22, ddd (9.5, 2.0, 2.0)	75.2	3.29	75.6	3.39, dd (10.0, 3.0)
3-OH		5.09, br d(2.0)				
4	27.9	1.74, m	28.8	1.94	28.2	1.75, m
5	14.4	0.72, d (7.0)	19.6	0.87	20.0	0.83, d (6.5)
6	20.2	0.74, d (7.0)	17.2	0.84	15.0	0.83, d (6.5)

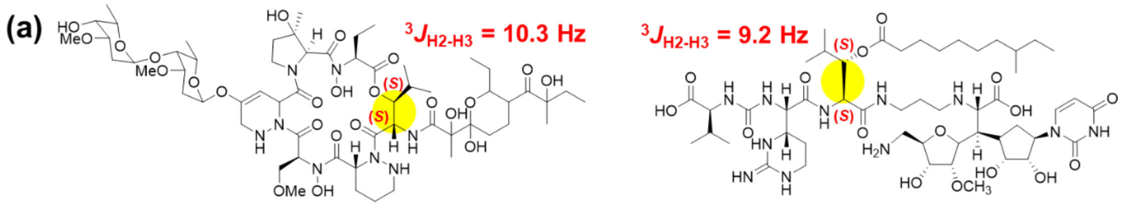

Mollemycin   Muramycin B$_1$

Compounds	Mollemycin[d]		Muramycin B1[e]	
Position	δC	δH (J in Hz)	δC	δH (J in Hz)
1	171.1		169.2	
2	56.1	4.90, dd (10.4, 10.3)	53.1	4.45, dd (9.2, 8.7)
2-NH		8.23, d (10.4)		8.45, d (8.7)
3	76.8	5.38, dd (10.3, 1.9)	75.0	4.93, dd (9.2, 3.3)
3-OH		-		-
4	29.5	1.87, m	27.8	1.91, m
5	21.0	0.94, d (6.9)	19.6	0.82
6	15.0	0.86, d (6.9)	15.7	0.80

(b)

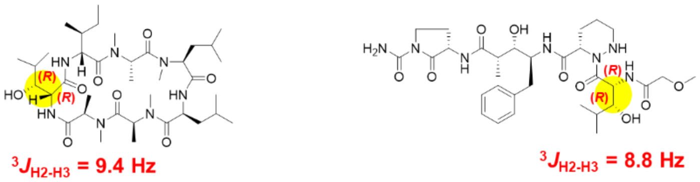

(-)-Ternatin Actinoramide A

Compounds	(-)-Ternatin[f]		Actinoramide A[g]	
Position	δC	δH (J in Hz)	δC	δH (J in Hz)
1	174.8		173.0	
2	56.3	5.17, dd (9.4, 8.0)	50.4	5.50, dd (8.8, 8.8)
2-NH		7.92, d (8.0)		7.43, d (8.8)
3	76.0	3.96, dd (9.4, 2.1)	75.7	3.43*
3-OH		5.87, s		4.79, d (7.1)
4	29.5	2.21-2.25, m	29.8	1.64, m
5	21.0	1.44, d (6.8)	20.8	0.87, d (7.1)
6	15.0	1.52, d (6.8)	16.1	0.83, d (7.1)

[a] NMR data were acquired in 800 MHz, DMSO-d_6
[b] NMR data were acquired in 500 MHz, DMSO-d_6
[c] NMR data were acquired in 600 MHz, DMSO-d_6
[d] NMR data were acquired in 900 MHz, $CDCl_3$
[e] NMR data were acquired in 500 MHz, DMSO-d_6
[f] NMR data were acquired in 800 MHz, C_6D_6
[g] NMR data were acquired in 500 MHz for ^{1}H and 75 MHz for ^{13}C, DMSO-d_6
* Overlapped

(c)

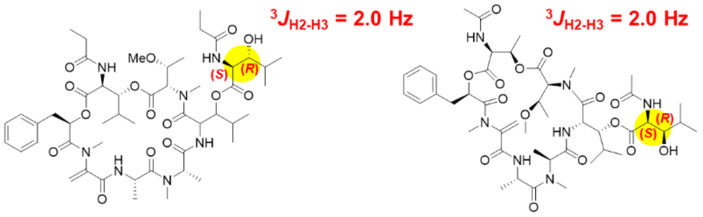

Sameuramide YM-254890

Compounds	Sameuramide[h]		YM-254890[i]	
Position	δC	δH (J in Hz)	δC	δH (J in Hz)
1	171.4		170.7	
2	57.9	4.36, dd (7.8, 2.0)	57.6	4.39, dd (7.9, 2.0)
2-NH		7.27, d (7.8)		7.17, d (7.9)
3	79.0	3.57, m	78.7	3.67, m
3-OH		6.91, d(4.4)		6.76, d (3.9)
4	31.0	1.93, m	30.6	1.96, m
5	18.7	0.84, d (6.8)	20.6	1.16, d (6.3)
6	20.8	1.16, d (6.6)	18.6	0.86, d (6.8)

(d)

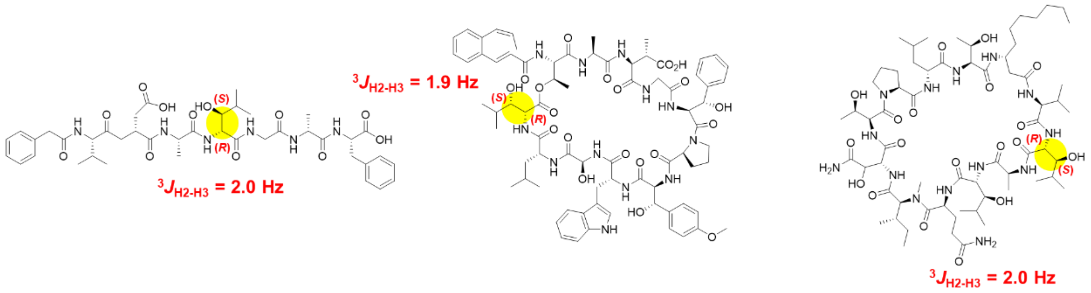

Compounds	JBIR-78[j]		Skyllamycin A[k]		Laxaphycin B[l]	
Position	δC	δH (J in Hz)	δC	δH (J in Hz)	δC	δH (J in Hz)
1	168.4		170.8		171.4	
2	55.8	4.29, dd (8.2, 2.0)	56.1	4.45, d (1.9)	55.2	4.34, dd (9.1, 2.0)
2-NH		7.89, d (8.2)				7.94, d (8.1)
3	76.3	3.53, dd (14.4, 2.0)	76.5	3.79, dd (9.2, 1.8)	76.4	3.49
3-OH						
4	27.9	1.56, dqq (14.4, 6.5, 6.5)	32.3	1.67, m	30.5	1.58
5	19.5	0.89, d (6.5)	19.2	1.02, d (6.7)	19.2	0.89
6	19.1	0.72, d (6.5)	18.7	0.83, d (6.7)	18.6	0.76

[h] NMR data were acquired in 600 MHz, $CDCl_3$
[i] NMR data were acquired in 500 MHz, dioxane-d_8
[j] NMR data were acquired in 600 MHz, DMSO-d_6
[k] NMR data were acquired in 500 MHz, CD_3OD
[l] NMR data were acquired in 500 MHz, DMSO

Table S3. Experimental and calculated chemical shifts of the partial structure of the epoxinnamide (**1**).

Position	Experimental chemical shifts	Calculated chemical shifts of 1a	Calculated chemical shifts of 1b
C-51	169.2	161.7	162.1
C-52	124.6	129.2	129.6
C-53	136.7	144.8	143.2
C-54	134.1	139.3	140.4
C-55	126.9	127.6	128.8
C-56	127.8	127.4	127.9
C-57	129.6	127.2	126.8
C-58	124.4	130.7	131.0
C-59	136.7	139.1	138.8
C-60	54.8	60.2	61.0
C-61	58.9	59.4	59.2
C-62	17.6	22.4	22.3
H-52	6.94	4.95	5.08
H-53	8.11	7.29	7.26
H-55	7.42	6.47	6.52
H-56	7.00	6.67	6.67
H-57	7.33	6.79	6.77
H-58	7.09	6.97	6.86
H-60	4.07	2.78	2.86
H-61	2.97	2.04	2.27
H_3-62a	1.37	0.65	0.73
H_3-62b	1.37	0.92	1.18
H_3-62c	1.37	1.06	0.80

Figure S16. Results of DP4 calculation for the partial structure of the epoxinnamide (**1**).

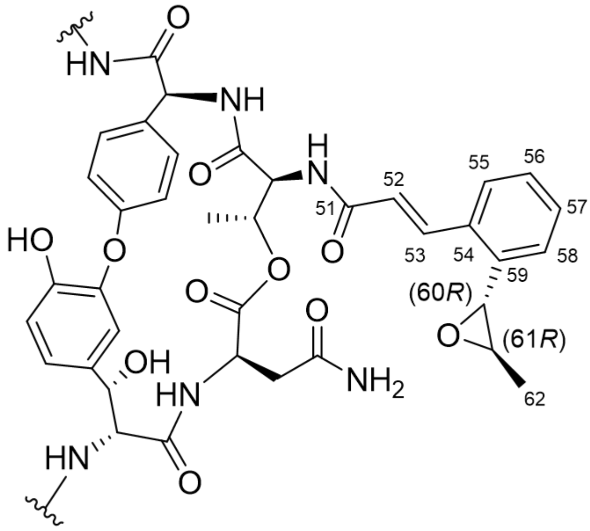

Isomer 1a

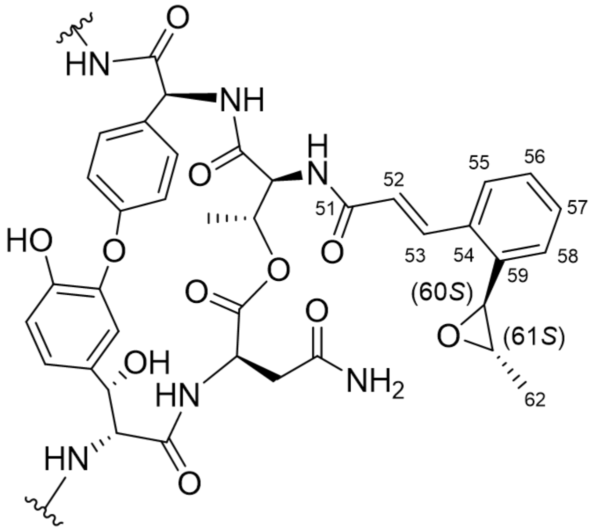

Isomer 1b

Results of DP4 using both carbon and proton data:<br>Isomer 1a: 0.3%<br>Isomer 1b: 99.7%	
**Results of DP4 using the carbon data only:**<br>Isomer 1a: 39.3%<br>Isomer 1b: 60.7%	**Results of DP4 using the proton data only:**<br>Isomer 1a: 0.5%<br>Isomer 1b: 99.5%

Table S4. AntiSMASH output table of the *Streptomyces* sp. OID44.

Region	Type	From	To	Most similar known cluster		Similarity
Region 1	NRPS	2	50,236	mycotrienin I	NRP + Polyketide	7%
Region 2	PKS-like, terpene	277,029	323,778	rustmicin	Polyketide: Iterative type I	20%
Region 3	terpene	454,797	476,446	tiancilactone	Terpene	17%
Region 4	NRPS	506,589	583,445	dechlorocuracomycin	NRP	8%
Region 5	RiPP-like	588,129	595,973			
Region 6	T1PKS,hglE-KS	603,812	653,884	leinamycin	NRP + Polyketide: Modular type I + Polyketide: Trans-AT type I	2%
Region 7	NRPS-like	691,682	732,097			
Region 8	T1PKS,NRPS	793,093	899,371	meridamycin	NRP + Polyketide	52%
Region 9	terpene	1,069,327	1,089,448	2-methylisoborneol	Terpene	100%
Region 10	terpene	1,192,137	1,211,829	pristinol	Terpene	100%
Region 11	NRPS-like,T1PKS	1,667,210	1,710,563	amipurimycin	Polyketide	90%
Region 12	T1PKS	1,917,850	2,000,720	lydicamycin	NRP + Polyketide: Modular type I	28%
Region 13	lanthipeptide-class-iii	2,100,735	2,123,356	lipopolysaccharide	Saccharide: Lipopolysaccharide	5%
Region 14	butyrolactone	2,470,261	2,479,489			
Region 15	blactam	2,497,249	2,520,539	valclavam / (-)-2-(2-hydroxy-ethyl)clavam	Other: Non-NRP beta-lactam	57%
Region 16	transAT-PKS,NRPS-like	2,650,736	2,727,582	cycloheximide	Polyketide: Trans-AT type I	50%
Region 17	T1PKS,siderophore	2,965,637	3,019,200	apoptolidin	Polyketide	23%
Region 18	ectoine	3,188,487	3,198,891	ectoine	Other	100%
Region 19	terpene	3,668,388	3,688,250			
Region 20	lanthipeptide-class-i	3,889,210	3,914,169			
Region 21	T1PKS	4,238,573	4,414,560	mediomycin A	Polyketide	56%
Region 22	RRE-containing	4,543,122	4,563,992	granaticin	Polyketide: Type II	10%
Region 23	ladderane	4,576,517	4,616,468	atratumycin	NRP	31%
Region 24	NRPS	4,661,153	4,704,484	ochronotic pigment	Other	75%
Region 25	indole	4,904,436	4,925,581	5-isoprenylindole-3-carbox-ylate β-D-glycosyl ester	Other	61%
Region 26	NRPS, arylpolyene, ladderane	5,206,961	5,308,896	atratumycin	NRP	57%
Region 27	terpene	5,523,184	5,543,637	geosmin	Terpene	100%

Region 28	siderophore	6,549,359	6,560,846	desferrioxamin B	Other	100%
Region 29	terpene	6,848,342	6,871,979	carotenoid	Terpene	63%
Region 30	NRPS-like	7,064,128	7,106,889	echoside A / echoside B / echoside C / echoside D / echoside E	NRP	100%
Region 31	siderophore	7,714,788	7,726,701			
Region 32	RiPP-like	7,940,469	7,951,776			
Region 33	redox-cofactor	8,080,073	8,100,903			
Region 34	T2PKS	8,180,420	8,252,935	spore pigment	Polyketide	83%
Region 35	betalactone	8,326,913	8,359,347			
Region 36	terpene	8,526,395	8,552,921	hopene	Terpene	76%
Region 37	lanthipeptide-class-i	8,739,188	8,763,626	steffimycin D	Polyketide: Type II + Saccharide: Hybrid/tailoring	16%
Region 38	other	8,769,851	8,811,938	mitomycin	Other: Aminocoumarin	18%
Region 39	NRPS	8,815,104	8,892,129	atratumycin	NRP	10%
Region 40	T1PKS	8,980,397	9,028,143			
Region 41	butyrolactone	9,200,735	9,211,667			
Region 42	hserlactone	9,222,163	9,242,921	daptomycin	NRP	3%
Region 43	redox-cofactor	9,374,199	9,396,401	lankacidin C	NRP + Polyketide	13%
Region 44	T1PKS	9,428,122	9,502,432	elaiophylin	Polyketide	87%
Region 45	T1PKS	9,638,180	9,768,523	nigericin	Polyketide: Modular type I	100%
Region 46	T1PKS	9,938,825	10,085,447	niphimycins C-E	Polyketide	87%
Region 47	NRPS-like	10,202,198	10,242,871	BD-12	NRP	67%
Region 48	NRPS	10,322,317	10,373,224	coelichelin	NRP	90%
Region 49	CDPS	10,398,124	10,418,870	bicyclomycin	Other: tRNA-derived	100%

Table S5. Deduced functions of ORFs in the epoxinnamide (**1**) biosynthetic gene cluster from the *Streptomyces* sp. OID44 [21].

ORF	Size (aa)	Annotation based on BLASTP			Homologues from *dml* gene cluster	
		Proposed function	Accession number	Identity	Homo-logues	Identity
orf1	492	trehalose-6-phosphate synthase	WP_210565462.1	100%		
orf2	288	trehalose phosphatase	KUL65503.1	99%		
orf3	100	DUF3263 domain-containing protein	WP_236669589.1	100%		
orf4	427	extracellular solute-binding protein	WP_210565464.1	100%		
orf5	319	glucokinase	SEC11617.1	99%		
orf6	394	N-acetylglucosamine-6-phosphate deacetylase	WP_059142728.1	100%		
orf7	320	1-phosphofructokinase family hexose kinase	WP_059142729.1	99%		
orf8	328	carbohydrate-binding protein	WP_237516519.1	98%		
orf9	562	diguanylate cyclase CdgB	WP_059142731.1	100%		
orf10	166	flavin reductase family protein	WP_079059208.1	100%		
orf11	130	aminoacyl-tRNA hydrolase	WP_059142733.1	100%		
orf12	191	TerD family protein	WP_059142734.1	100%		
orf13	198	Uma2 family endonuclease	WP_205587732.1	99%		
orf14	193	TetR family transcriptional regulator	WP_093462714.1	95%		
orf15	336	NAD(P)-dependent alcohol dehydrogenase	WP_086706493.1	100%		
orf16	539	M4 family metallopeptidase	WP_086706492.1	100%		
orf17	361	S-(hydroxymethyl)mycothiol dehydrogenase	WP_059142739.1	100%		
orf18	212	MBL fold metallo-hydrolase	WP_086706490.1	100%	*orf(-2)*	95%
orf19	169	flavin reductase family protein	WP_059142741.1	100%	*orf(-1)*	88%
epcA	4,775	non-ribosomal peptide synthetase	WP_059142742.1	100%	*dmlA*	78%
orf20	204	hypothetical protein	WP_086710114.1	100%		
epcB	421	cytochrome P450	WP_125755238.1	100%	*dmlC*	95%
epcC	3,905	non-ribosomal peptide synthetase	WP_210565471.1	100%	*dmlD*	83%
epcD	1,610	non-ribosomal peptide synthetase	WP_059142746.1	99%	*dmlE*	87%
epcE	2,077	non-ribosomal peptide synthetase	WP_125755234.1	100%	*dmlE*	80%
epcF	405	cytochrome P450	WP_059142747.1	99%	*dmlF*	95%
epcG	357	LLM class flavin-dependent oxidoreductase	WP_059142748.1	100%	*dmlG*	91%

orf21	264	ABC transporter permease	WP_059142749.1	100%	*orf5*	95%
orf22	324	ABC transporter	KUL65527.1	100%	*orf6*	94%
epcH	408	cytochrome P450	WP_125755230.1	100%	*dmlH*	93%
epcI	347	*p*-hydroxymandelate synthase	UNJ19134.1	91%	*dmlI*	91%
epcJ	824	*p*-hydroxyphenylglycine aminotransferase/ *p*-hydroxymandelate oxidase fusion protein	UNJ19135.1	94%	*dmlJ*	81%
epcK	369	prephenate dehydrogenase	WP_059142753.1	100%	*dmlK*	94%
orf23	696	AarF/UbiB family protein	WP_059142754.1	100%	*orf17*	93%
orf24	68	MbtH family protein	WP_044573519.1	100%	*orf18*	94%
epcL	237	Isomerase	MYV58306.1	77%	*dmlL*	89%
epcM	248	3-oxoacyl-[acyl-carrier-protein] reductase	WP_086710106.1	100%	*dmlM*	95%
epcN	164	beta-hydroxyacyl-ACP dehydratase	WP_059142757.1	99%	*dmlN*	90%
epcO	147	dehydratase	KUL65536.1	100%		
epcP	375	3-oxoacyl-ACP synthase	WP_059142759.1	100%	*dmlO*	96%
orf25	339	hypothetical protein	WP_125755222.1	100%	*orf20*	97%
orf26	307	alpha/beta hydrolase	WP_125755219.1	100%	*orf21*	91%
epcQ	314	3-oxoacyl-ACP synthase	WP_059142762.1	100%	*dmlP*	91%
epcR	377	3-oxoacyl-ACP synthase	WP_059142763.1	100%	*dmlQ*	96%
epcS	413	beta-ketoacyl-[acyl-carrier-protein] synthase family protein	WP_059142764.1	100%	*dmlR*	97%
epcT	83	acyl carrier protein	WP_014061562.1	100%	*dmlS*	99%

Figure S17. Biosynthetic functions of the cytochrome P450s and the thioesterase domains in epoxinnamide (**1**), nyuzenamide C, skyllamycin A, atratumycin, and telomycin [15,21,33,34].

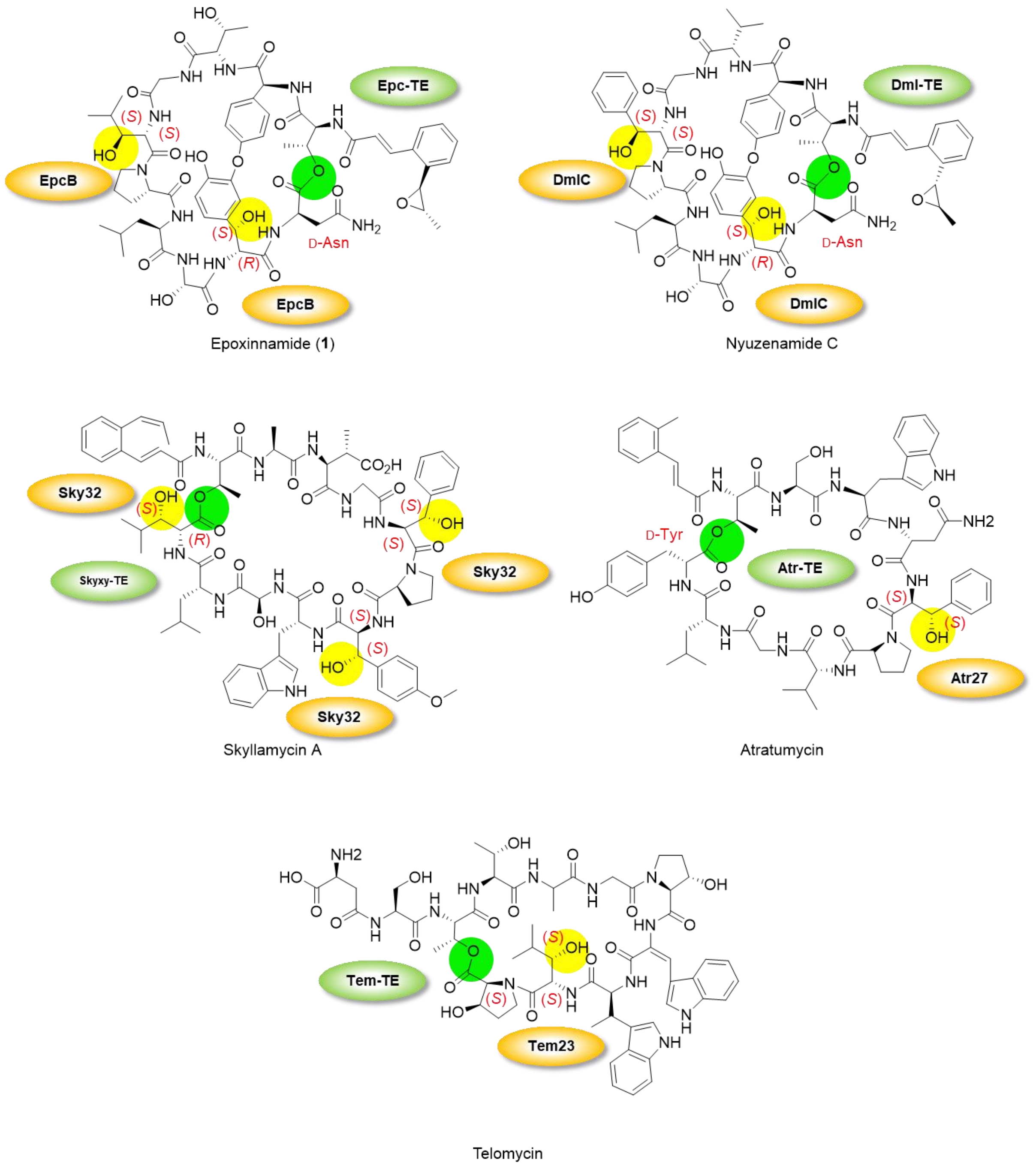

Figure S18. Sequence alignment of the Epc-TE with the other TEs [35]. (**a**) Full sequence alignments of the thioesterase domains, (**b**) Important active site residues are highlighted in the red boxes. Each shaded color means the similarity between sequences. Black = 100% similarity, dark gray = 80 to 100% similarity, gray = 60 to 80% similarity, white = less than 60% similarity.

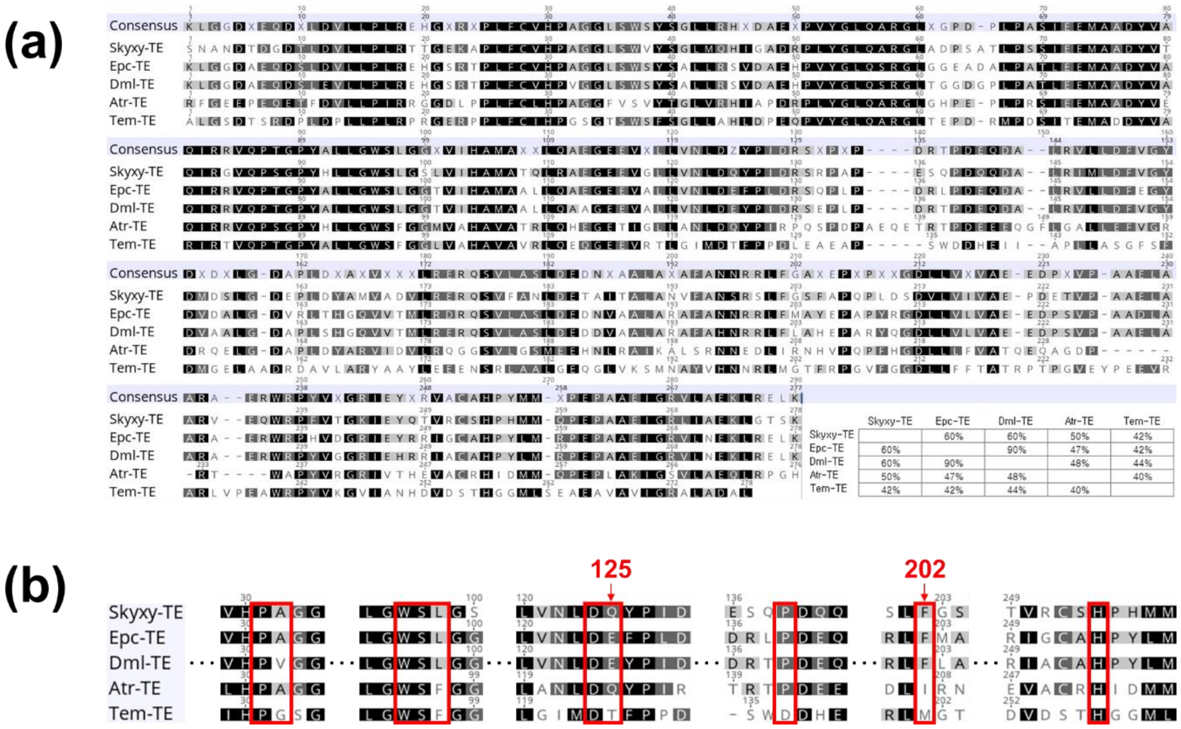

	Skyxy-TE	Epc-TE	Dml-TE	Atr-TE	Tem-TE
Skyxy-TE		60%	60%	50%	42%
Epc-TE	60%		90%	47%	42%
Dml-TE	60%	90%		48%	44%
Atr-TE	50%	47%	48%		40%
Tem-TE	42%	42%	44%	40%	

Figure S19. Proposed structural models of thioesterase domains, active site residues surrounding catalytic Ser residue, and key active sites for dual function of (**a**) Epc-TE, (**b**) Dml-TE, (**c**) Atr-TE, and (**d**) Tel-TE. Epc-TE, Dml-TE, and Atr-TE have homologous active site structures.

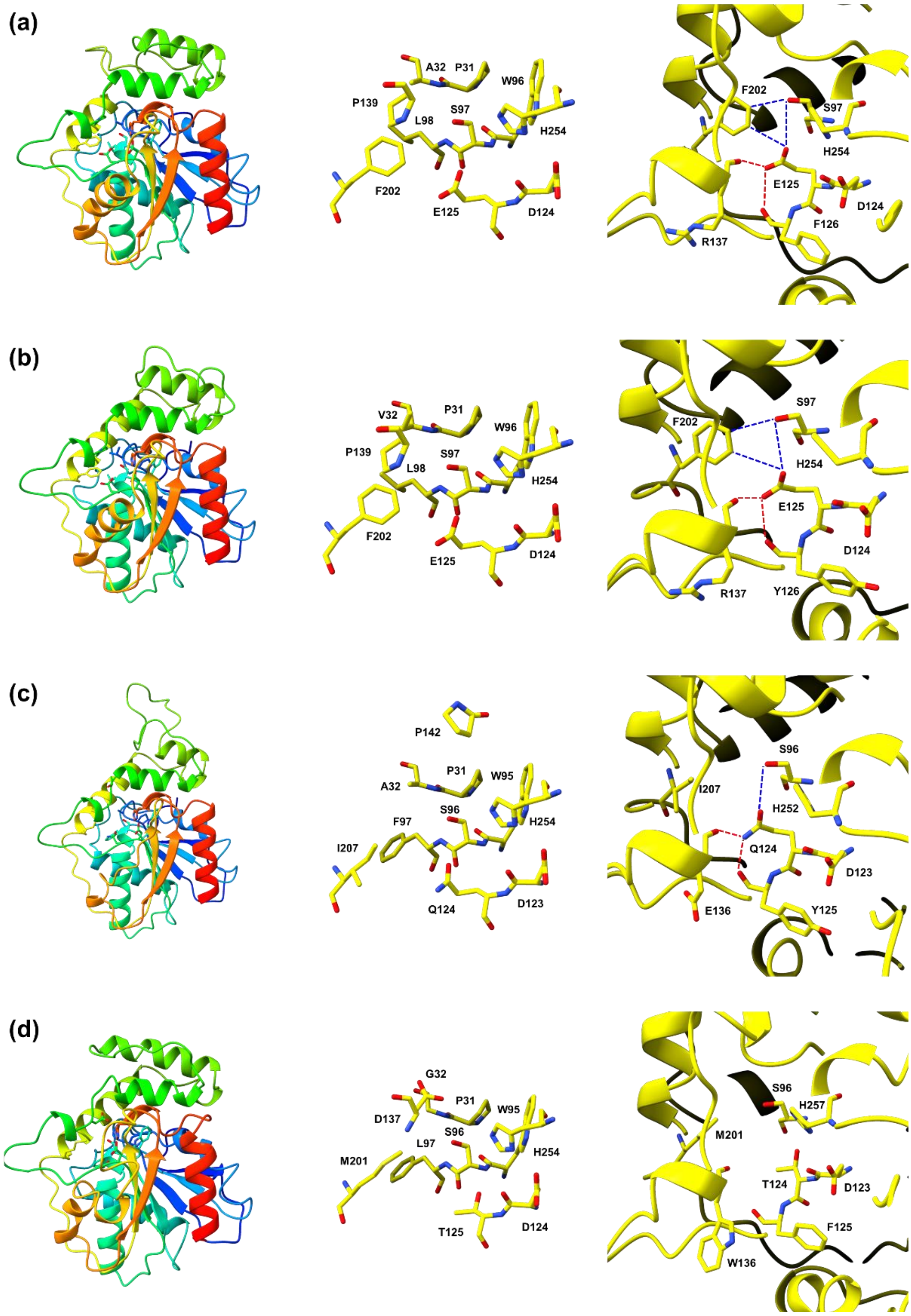

Figure S20. Sequence alignment of the EpcB with the other cytochrome P450s [15,21,33,34]. Each shaded color means the similarity between sequences. Black = 100% similarity, dark gray = 80 to 100% similarity, gray = 60 to 80% similarity, white = less than 60% similarity.

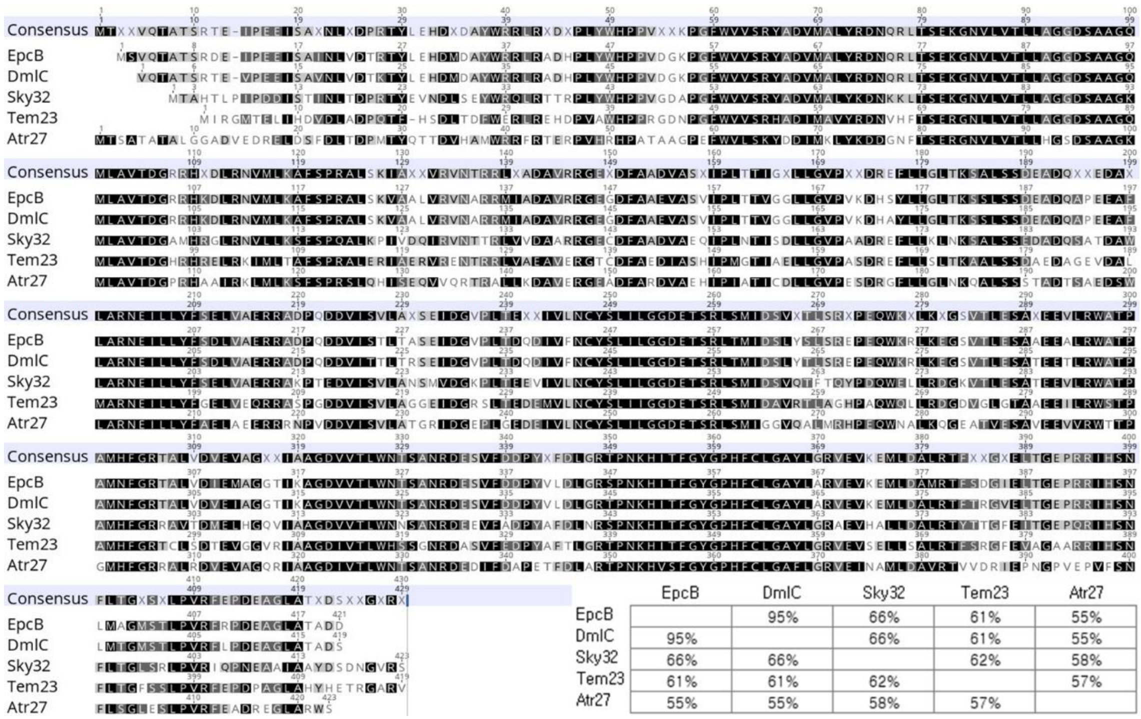

	EpcB	DmlC	Sky32	Tem23	Atr27
EpcB		95%	66%	61%	55%
DmlC	95%		66%	61%	55%
Sky32	66%	66%		62%	58%
Tem23	61%	61%	62%		57%
Atr27	55%	55%	58%	57%	

Figure S21. Phylogenetic analysis of the cytochrome P450 β-hydroxylases which catalyze β-hydroxylation of amino acid. Amino acid sequences were collected from MIBiG (https://mibig.secondarymetabolites.org/, accessed on 12 July 2022) and NCBI (https://www.ncbi.nlm.nih.gov/protein/, accessed on 12 July 2022). Phylogenetic tree was built by Geneious Tree Builder. Cytochrome P450 monooxygenase CYP142 from *Mycobacterium tuberculosis* H37Rv (NP_218035) was used as an out group (not shown). Branch labels represent the consensus support (%) estimated by bootstrap analysis with 1,000 replicates (support threshold 50%). The scale bar indicates the average number of substitutions per site. R = amino acid residues. Orf8 catalyzes *S*-hydroxylation of leucine unit, opposed to its position in the phylogenetic tree. Stereochemical information of NikQ and SanQ are not known. Cytochrome P450 monooxygenase for *β*-hydroxylation could be largely divided into two groups, 3*S*-hydroxylase and 3*R*-hydroxylase but there were some exceptions: NovI and OxyD are representative 3*R*-hydroxylase which catalyze *β*-hydroxylation of tyrosine residues in biosynthesis of aminocoumarin and glycopeptide antibiotics, respectively. However, these two enzymes belong to different clade in the phylogenetic tree. This observation decreases the reliability to deduce OH stereochemistry with the sequences of hydroxylases. Nonetheless, Sky32, EpcB, Tem23 and Atr27 belong to the 3*S*-hydroxylase clade. Especially, EpcB and DmlC are phylogenetically closely related to Sky32 which catalyzes *S*-stereoselective *β*-hydroxylation in PCP domain-dependent manner.

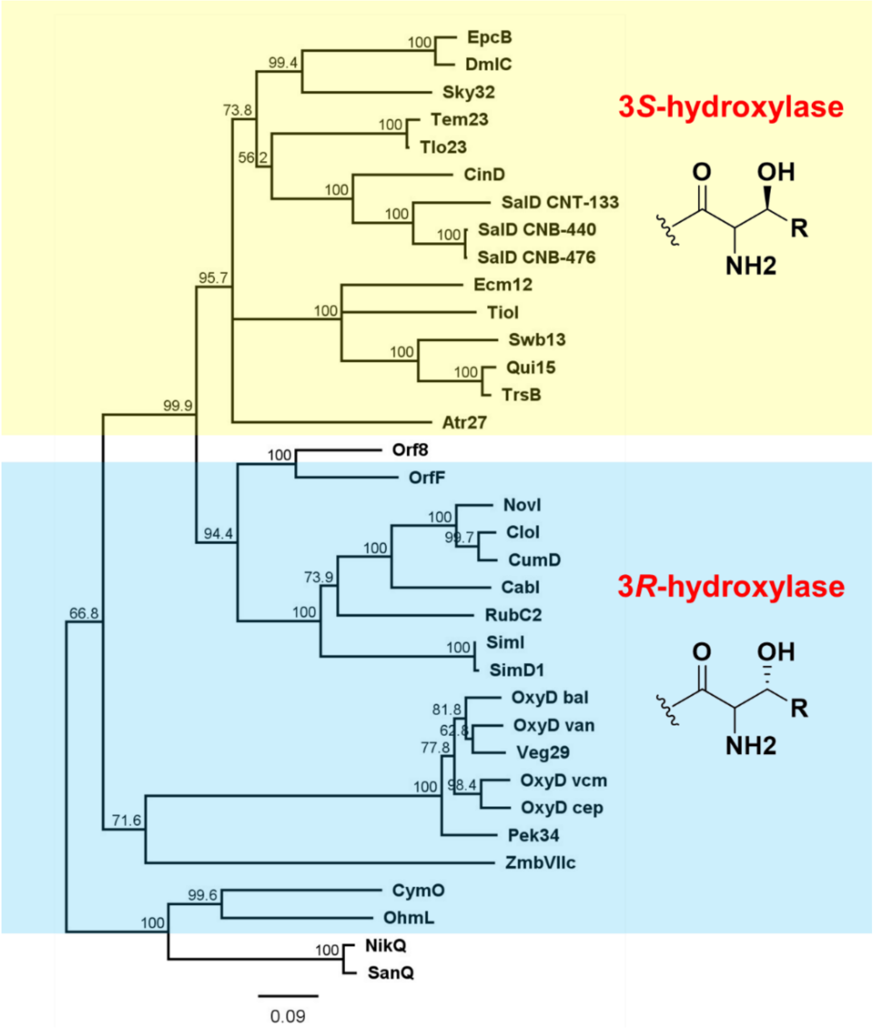

Table S6. List of the cytochrome P450 *β*-hydroxylases implicated in the *β*-hydroxylation of amino acid residues. Amino acid substrates and final products are shown in the table.

CYP450	Accession Num	Amino acid substrates	Final product	Stereo-chemistry
EpcB	-	Tyr, Leu	Epoxinnamide	3*S*
DmlC	UNJ19128	Tyr, Phe	Nyuzenamide	3*S*
Sky32	AEA30275	Phe, OMe-Tyr, Leu	Skyllamycin	3*S*
Tem23	AKQ13298	Leu	Telomycin	3*S*
Tlo23	ALV86869	Leu	Telomycin	3*S*
CinD	CBW54674	3-cyclohex-2′-enylalanine	Cinnabaramide	3*S*
SalD CNT-133	ADZ28490	3-cyclohex-2′-enylalanine	Salinosporamide	3*S*
SalD CNB-440	ABP53495	3-cyclohex-2′-enylalanine	Salinosporamide	3*S*
SalD CNB-476	ABP73648	3-cyclohex-2′-enylalanine	Salinosporamide	3*S*
Ecm12	BAE98161	Trp	Echinomycin	3*S*
TioI	CAJ34365	Trp	Thiocoraline	3*S*
Swb13	BAI63285	Trp	SW-163C	3*S*
Qui15	AET98913	Trp	Quinomycin	3*S*
TrsB	BAH04170	Trp	Triostin	3*S*
Atr27	QBG38788	Phe	Atratumycin	3*S*
Orf8	BAX90002	Leu	JBIR-78	3*S*
OrfF	WP_067444944	Leu	Cysteoamide	3*R*
NovI	AAF67502	Tyr	Novobiocin	3*R*
CloI	AAN65225	Tyr	Clorobiocin	3*R*
CumD	AAG29781	Tyr	Coumermycin	3*R*
CabI	ACU71637	Tyr	Cacibiocin	3*R*
RubC2	CAI94719	Tyr	Rubradirin	3*R*
SimI	AAG34185	Tyr	Simocyclinone	3*R*
SimD1	AAK06805	Tyr	Simocyclinone	3*R*
OxyD bal	CAC48370	Tyr	Balhimycin	3*R*
OxyD van	CAA11772	Tyr	Vancomycin	3*R*
Veg29	ACJ60971	Tyr	VEG type I glycopeptide antibiotic	3*R*
OxyD vcm	AEI58880	Tyr	Vancomycin	3*R*
OxyD cep	CCD33151	Tyr	Chloroeremomycin	3*R*
Pek34	AGF91769	Tyr	Pekiskomycin	3*R*
ZmbVIIc	ACG60779	Val	Zorbamycin	-
CymO	ABW00322	Phe	Cyclomarin	3*R*
OhmL	QGA70159	Phe	Ohmyungsamycin	3*R*
NikQ	CAB75339.1	His	Nikkomycin	Unknown
SanQ	AAG48135	His	Nikkomycin	Unknown

Figure S22. Phylogenetic tree and table of the ketosynthases in BGC of youssoufene and CCNPs (atratumycin, cinnapeptin, nyuzenamide C, epoxinnamide, kitacinnamycin, skyllamycin A, WS9326A, pepticinnamin E, and coprisamide C) [36,37].

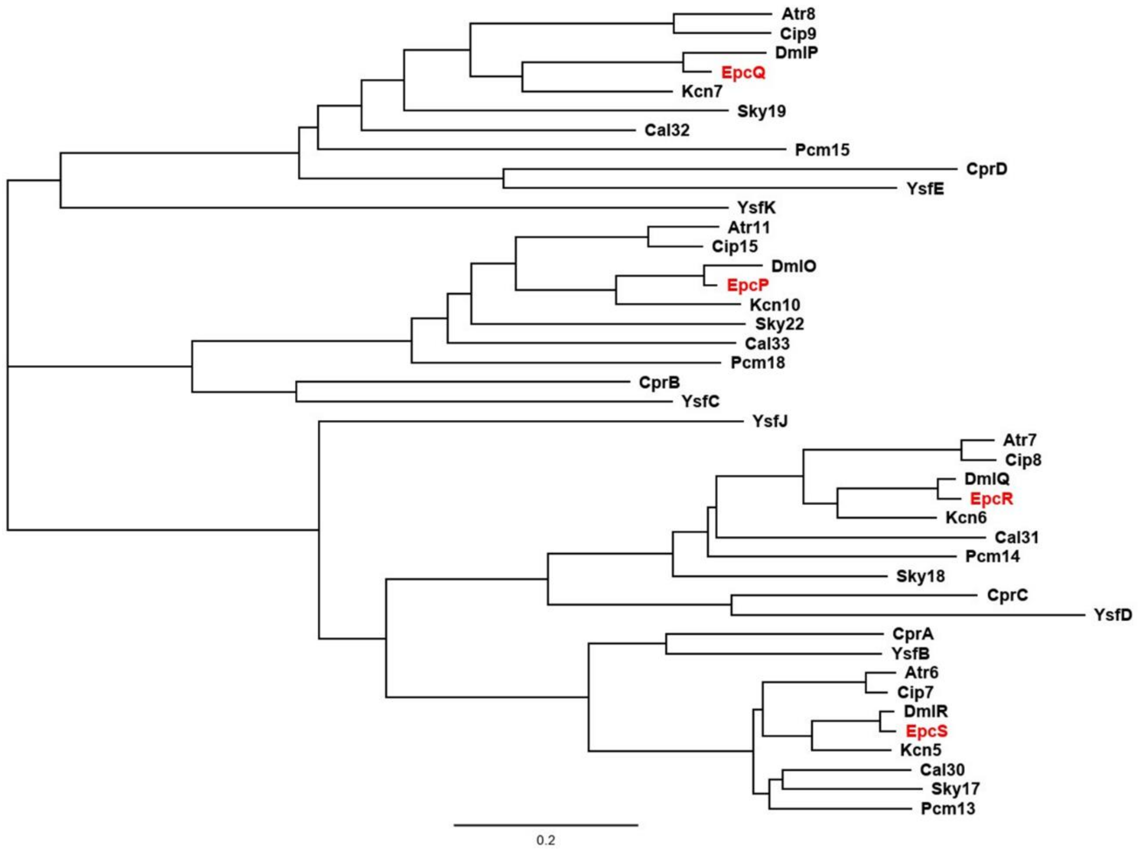

Compounds	KS_1	CLF_1	KS_2	CLF_2	KS_3	CLF_3
Youssoufene	YsfB	YsfC	YsfD	YsfE	YsfJ	YsfK
Atratumycin	Atr6	Atr11	Atr7	Atr8		
Cinnapeptin	Cip7	Cip15	Cip8	Cip9		
Nyuzenamide C	DmlR	DmlO	DmlQ	DmlP		
Epoxinnamide	EpcS	EpcP	EpcR	EpcQ		
Kitacinnamycin	Kcn5	Kcn10	Kcn6	Kcn7		
Skyllamycin A	Sky17	Sky22	Sky18	Sky19		
WS9326A	Cal30	Cal33	Cal31	Cal32		
Pepticinnamin E	Pcm13	Pcm18	Pcm14	Pcm15		
Coprisamide C	CprA	CprB	CprC	CprD		

Table S7. Quinone reductase assay data of nyuzenamide C and epoxinnamide (**1**).

Sample	Concentration	Average	Standard Deviation
Control	0	1	0.071686
Nyuzenamide C (μM)	20	1.644478	0.152603
	10	1.390253	0.157468
	5	1.126734	0.035390
Epoxinnamide (μM)	5	1.605825	0.098615
	2.5	1.357639	0.099641
	1.25	1.170649	0.072016
β-naphthoflavone (μM)	2	2.213906	0.021521

Table S8. Cell viability data of nyuzenamide C and epoxinnamide (**1**).

Sample	Concentration	Average	Standard Deviation
Control	0	1	0.125867
Nyuzenamide C (μM)	20	0.943992	0.071223
	10	1.041755	0.026192
	5	1.089161	0.065328
Epoxinnamide (μM)	5	0.963808	0.084109
	2.5	0.975457	0.067945
	1.25	1.054388	0.033515
β-naphthoflavone (μM)	2	0.998161	0.012416

Table S9. In vitro capillary tube formation assay data of nyuzenamide C and epoxinnamide (**1**).

Concentration (μM)	Average of Total Segments Length	Tube Formation (%)
VEGF +	5223.67	100
VEGF −	2307.22	44.17
Nyuzenamide C, 20	2625.02	50.25
Nyuzenamide C, 10	3715.86	71.14
Nyuzenamide C, 5	4877.50	93.37
Epoxinnamide, 20	3219.53	61.63
Epoxinnamide, 10	4180.46	80.03
Epoxinnamide, 5	5029.67	96.29
Sunitinib, 4	2943.54	56.35
Sunitinib, 2	3595.32	68.83
Sunitinib, 1	4538.97	86.89

References

1. Fischbach, M.A.; Walsh, C.T. Assembly-Line Enzymology for Polyketide and Nonribosomal Peptide Antibiotics: Logic, Machinery, and Mechanisms. *Chem. Rev.* **2006**, *106*, 3468–3496, doi:10.1021/cr0503097.
2. Hayashi, K.; Hashimoto, M.; Shigematsu, N.; Nishikawa, M.; Ezaki, M.; Yamashita, M.; Kiyoto, S.; Okuhara, M.; Kohsaka, M.; Imanaka, H. WS9326A, a Novel Tachykinin Antagonist Isolated from *Streptomyces violaceusniger* No. 9326. I. Taxonomy, Fermentation, Isolation, Physico-chemical Properties and Biological Activities. *J. Antibiot. (Tokyo)* **1992**, *45*, 1055–1063, doi.org/10.7164/antibiotics.45.1064.
3. Zhang, S.; Zhu, J.; Zechel, D.L.; Jessen-Trefzer, C.; Eastman, R.T.; Paululat, T.; Bechthold, A. New WS9326A Derivatives and One New Annimycin Derivative with Antimalarial Activity are Produced by *Streptomyces asterosporus* DSM 41452 and Its Mutant. *Chembiochem* **2018**, 19, 272–279, doi.org/10.1002/cbic.201700428.
4. Yu, Z.; Vodanovic-Jankovic, S.; Kron, M.; Shen, B. New WS9326A Congeners from *Streptomyces* sp. 9078 Inhibiting *Brugia malayi* Asparaginyl-tRNA Synthetase. *Org. Lett. 14*, 4946–4949, doi.org/10.1021/ol302298k.
5. Bae, M.; Oh, J.; Bae, E.S.; Oh, J.; Hur, J.; Suh, Y.-G.; Lee, S.K.; Shin, J.; Oh, D.-C. WS9326H, an Antiangiogenic Pyrazolone-Bearing Peptide from an Intertidal Mudflat Actinomycete. *Org. Lett.* **2018**, *20*, 1999–2002, doi:10.1021/acs.orglett.8b00546.
6. Shiomi, K.; Yang, H.; Inokoshi, J.; Van der Pyl, D.; Nakagawah, A.; Takeshima, H.; Omura, S. Pepticinnamins, New Farnesyl-protein Transferase Inhibitors Produced by an Actinomycete. II. Structural Elucidation of Pepticinnamin E. *J. Antibiot. (Tokyo)* **1993**, *46*, 229–234, doi.org/10.7164/antibiotics.46.229.
7. Toki, S.; Agatsuma, T.; Ochiai, K.; Saitoh, Y.; Ando, K.; Nakanishi, S.; Lokker, N.A.; Giese, N.A.; Matsuda, Y. RP-1776, a Novel Cyclic Peptide Produced by *Streptomyces* sp., inhibits the Binding of PDGF to the Extracellular Domain of Its Receptor. *J. Antibiot.* **2001**, *54*, 405–414. https://doi.org/10.7164/antibiotics.54.405.
8. Pohle, S.; Appelt, C.; Roux, M.; Fiedler, H.-P.; Süssmuth, R.D. Biosynthetic Gene Cluster of the Non-Ribosomally Synthesized Cyclodepsipeptide Skyllamycin: Deciphering Unprecedented Ways of Unusual Hydroxylation Reactions. *J. Am. Chem. Soc.* **2011**, *133*, 6194–6205, doi:10.1021/ja108971p.
9. Navarro, G.; Cheng, A.T.; Peach, K.C.; Bray, W.M.; Bernan, V.S.; Yildiz, F.H.; Linington, R.G. Image-Based 384-Well High-Throughput Screening Method for the Discovery of Skyllamycins A to C as Biofilm Inhibitors and Inducers of Biofilm Detachment in Pseudomonas Aeruginosa. *Antimicrob. Agents Chemother.* **2014**, *58*, 1092–1099, doi:10.1128/AAC.01781-13.
10. Bracegirdle, J.; Hou, P.; Nowak, V.V.; Ackerley, D.F.; Keyzers, R.A.; Owen, J.G. Skyllamycins D and E, Non-Ribosomal Cyclic Depsipeptides from Lichen-Sourced *Streptomyces anulatus*. *J. Nat. Prod.* **2021**, *84*, 2536–2543, doi:10.1021/acs.jnatprod.1c00547.
11. Zhang, F.; Adnani, N.; Vazquez-Rivera, E.; Braun, D.R.; Tonelli, M.; Andes, D.R.; Bugni, T.S. Application of 3D NMR for Structure Determination of Peptide Natural Products. *J. Org. Chem.* **2015**, *80*, 8713–8719, doi:10.1021/acs.joc.5b01486.
12. Zhang, C.; Seyedsayamdost, M.R. Discovery of a Cryptic Depsipeptide from *Streptomyces ghanaensis* via MALDI-MS-Guided High-Throughput Elicitor Screening. *Angew. Chem. Int. Ed.* **2020**, *132*, 23005–23009, doi:10.1002/anie.202009611.
13. Liu, M.; Liu, N.; Shang, F.; Huang, Y. Activation and Identification of NC-1: A Cryptic Cyclodepsipeptide from Red Soil-Derived *Streptomyces* sp. FXJ1.172. *European J. Org. Chem.* **2016**, *2016*, 3943–3948, doi:10.1002/ejoc.201600297.
14. Bae, M.; Kim, H.; Moon, K.; Nam, S.-J.; Shin, J.; Oh, K.-B.; Oh, D.-C. Mohangamides A and B, New Dilactone-Tethered Pseudo-Dimeric Peptides Inhibiting *Candida Albicans* Isocitrate Lyase. *Org. Lett.* **2015**, *17*, 712–715, doi:10.1021/ol5037248.
15. Sun, C.; Yang, Z.; Zhang, C.; Liu, Z.; He, J.; Liu, Q.; Zhang, T.; Ju, J.; Ma, J. Genome Mining of *Streptomyces atratus* SCSIO ZH16: Discovery of Atratumycin and Identification of Its Biosynthetic Gene Cluster. *Org. Lett.* **2019**, *21*, 1453–1457 , doi:10.1021/acs.orglett.9b00208.
16. Liu, Q.; Liu, Z.; Sun, C.; Shao, M.; Ma, J.; Wei, X.; Zhang, T.; Li, W.; Ju, J. Discovery and Biosynthesis of Atrovimycin, an Antitubercular and Antifungal Cyclodepsipeptide Featuring Vicinal-Dihydroxylated Cinnamic Acyl Chain. *Org. Lett.* **2019**, *21*, 2634–2638, doi:10.1021/acs.orglett.9b00618.
17. Shi, J.; Liu, C.L.; Zhang, B.; Guo, W.J.; Zhu, J.; Chang, C.-Y.; Zhao, E.J.; Jiao, R.H.; Tan, R.X.; Ge, H.M. Genome Mining and Biosynthesis of Kitacinnamycins as a STING Activator. *Chem. Sci.* **2019**, *10*, 4839–4846, doi.org/10.1039/C9SC00815B.
18. Um, S.; Park, S.H.; Kim, J.; Park, H.J.; Ko, K.; Bang, H.-S.; Lee, S.K.; Shin, J.; Oh, D.-C. Coprisamides A and B, New Branched Cyclic Peptides from a Gut Bacterium of the Dung Beetle *Copris tripartitus*. *Org. Lett.* **2015**, *17*, 1272–1275, doi:10.1021/acs.orglett.5b00249.
19. Shin, Y.-H.; Ban, Y.H.; Kim, T.H.; Bae, E.S.; Shin, J.; Lee, S.K.; Jang, J.; Yoon, Y.J.; Oh, D.-C. Structures and Biosynthetic Pathway of Coprisamides C and D, 2-Alkenylcinnamic Acid-Containing Peptides from the Gut

Bacterium of the Carrion Beetle *Silpha perforata*. *J. Nat. Prod.* **2021**, *84*, 239–246, doi:10.1021/acs.jnatprod.0c00864.
20. Karim, Md.R.U.; In, Y.; Zhou, T.; Harunari, E.; Oku, N.; Igarashi, Y. Nyuzenamides A and B: Bicyclic Peptides with Antifungal and Cytotoxic Activity from a Marine-Derived *Streptomyces* sp. *Org. Lett.* **2021**, *23*, 2109–2113, doi:10.1021/acs.orglett.1c00210.
21. An, J.S.; Kim, M.-S.; Han, J.; Jang, S.C.; Im, J.H.; Cui, J.; Lee, Y.; Nam, S.-J.; Shin, J.; Lee, S.K.; Yoon, Y.J.; Oh, D.-C. Nyuzenamide C, an Antiangiogenic Epoxy Cinnamic Acid-Containing Bicyclic Peptide from a Riverine *Streptomyces* sp. *J. Nat. Prod.* **2022**, *85*, 804–814, doi:10.1021/acs.jnatprod.1c00837.
22. Kumar, N.G.; Urry, D.W. Proton Magnetic Resonance Assignments of the Polypeptide Antibiotic Telomycin. *Biochemistry* **1973**, *12*, 3811–3817, doi:10.1021/bi00744a003.
23. Wyche, T.P.; Ruzzini, A.C.; Beemelmanns, C.; Kim, K.H.; Klassen, J.L.; Cao, S.; Poulsen, M.; Bugni, T.S.; Currie, C.R.; Clardy, J. Linear Peptides Are the Major Products of a Biosynthetic Pathway That Encodes for Cyclic Depsipeptides. *Org. Lett.* **2017**, *19*, 1772–1775, doi:10.1021/acs.orglett.7b00545.
24. Raju, R.; Khalil, Z.G.; Piggott, A.M.; Blumenthal, A.; Gardiner, D.L.; Skinner-Adams, T.S.; Capon, R.J. Mollemycin A: An Antimalarial and Antibacterial Glyco-Hexadepsipeptide-Polyketide from an Australian Marine-Derived *Streptomyces* sp. (CMB-M0244). *Org. Lett.* **2014**, *16*, 1716–1719, doi:10.1021/ol5003913.
25. McDonald, L.A.; Barbieri, L.R.; Carter, G.T.; Lenoy, E.; Lotvin, J.; Petersen, P.J.; Siegel, M.M.; Singh, G.; Williamson, R.T. Structures of the Muraymycins, Novel Peptidoglycan Biosynthesis Inhibitors. *J. Am. Chem. Soc.* **2002**, *124*, 10260–10261, doi:10.1021/ja017748h.
26. Shimokawa, K.; Mashima, I.; Asai, A.; Ohno, T.; Yamada, K.; Kita, M.; Uemura, D. Biological Activity, Structural Features, and Synthetic Studies of (−)-Ternatin, a Potent Fat-Accumulation Inhibitor of 3T3-L1 Adipocytes. *Chem. Asian J.* **2008**, *3*, 438–446, doi:10.1002/asia.200700243.
27. Nam, S.-J.; Kauffman, C.A.; Jensen, P.R.; Fenical, W. Isolation and Characterization of Actinoramides A–C, Highly Modified Peptides from a Marine Streptomyces sp. *Tetrahedron* **2011**, *67*, 6707–6712, doi:10.1016/j.tet.2011.04.051.
28. Machida, K.; Arai, D.; Katsumata, R.; Otsuka, S.; Yamashita, J.K.; Ye, T.; Tang, S.; Fusetani, N.; Nakao, Y. Sameuramide A, a New Cyclic Depsipeptide Isolated from an Ascidian of the Family Didemnidae. *Bioorg. Med. Chem.* **2018**, *26*, 3852–3857, doi:10.1016/j.bmc.2018.06.042.
29. Taniguchi, M.; Suzumura, K.; Nagai, K.; Kawasaki, T.; Takasaki, J.; Sekiguchi, M.; Moritani, Y.; Saito, T.; Hayashi, K.; Fujita, S.; Tsukamoto, S.-I.; Suzuki, K.-I. YM-254890 Analogues, Novel Cyclic Depsipeptides with Gαq/11 Inhibitory Activity from *Chromobacterium* sp. QS3666. *Bioorg. Med. Chem.* **2004**, *12*, 3125–3133, doi:10.1016/j.bmc.2004.04.006.
30. Takeda, K.; Kemmoku, K.; Satoh, Y.; Ogasawara, Y.; Shin-ya, K.; Dairi, T. *N* -Phenylacetylation and Nonribosomal Peptide Synthetases with Substrate Promiscuity for Biosynthesis of Heptapeptide Variants, JBIR-78 and JBIR-95. *ACS Chem. Biol.* **2017**, *12*, 1813–1819, doi:10.1021/acschembio.7b00314.
31. Giltrap, A.M.; Haeckl, F.P.J.; Kurita, K.L.; Linington, R.G.; Payne, R.J. Synthetic Studies Toward the Skyllamycins: Total Synthesis and Generation of Simplified Analogues. *J. Org. Chem.* **2018**, *83*, 7250–7270, doi:10.1021/acs.joc.8b00898.
32. Boyaud, F.; Mahiout, Z.; Lenoir, C.; Tang, S.; Wdzieczak-Bakala, J.; Witczak, A.; Bonnard, I.; Banaigs, B.; Ye, T.; Inguimbert, N. First Total Synthesis and Stereochemical Revision of Laxaphycin B and Its Extension to Lyngbyacyclamide A. *Org. Lett.* **2013**, *15*, 3898–3901, doi:10.1021/ol401645m.
33. Uhlmann, S.; Süssmuth, R.D.; Cryle, M.J. Cytochrome $P450_{sky}$ Interacts Directly with the Nonribosomal Peptide Synthetase to Generate Three Amino Acid Precursors in Skyllamycin Biosynthesis. *ACS Chem. Biol.* **2013**, *8*, 2586–2596, doi:10.1021/cb400555e.
34. Fu, C.; Keller, L.; Bauer, A.; Brönstrup, M.; Froidbise, A.; Hammann, P.; Herrmann, J.; Mondesert, G.; Kurz, M.; Schiell, M.; Schummer, D.; Toti, L.; Wink, J.; Müller, R. Biosynthetic Studies of Telomycin Reveal New Lipopeptides with Enhanced Activity. *J. Am. Chem. Soc.* **2015**, *137*, 7692–7705, doi:10.1021/jacs.5b01794.
35. Yu, J.; Song, J.; Chi, C.; Liu, T.; Geng, T.; Cai, Z.; Dong, W.; Shi, C.; Ma, X.; Zhang, Z.; Ma, X.; Xing, B.; Jin, H.; Zhang, L.; Dong, S.; Yang, D.; Ma, M. Functional Characterization and Crystal Structure of the Bifunctional Thioesterase Catalyzing Epimerization and Cyclization in Skyllamycin Biosynthesis. *ACS Catal.* **2021**, *11*, 11733–11741, doi:10.1021/acscatal.1c03064.
36. Deng, Z.; Liu, J.; Li, T.; Li, H.; Liu, Z.; Dong, Y.; Li, W. An Unusual Type II Polyketide Synthase System Involved in Cinnamoyl Lipid Biosynthesis. *Angew. Chem. Int. Ed.* **2021**, *133*, 153–158, doi:10.1002/anie.202007777.
37. Shi, J.; Shi, Y.; Li, J.C.; Wei, W.; Chen, Y.; Cheng, P.; Liu, C.L.; Zhang, H.; Wu, R.; Zhang, B.; Jiao, R.H.; Yu, S.; Liang, Y.; Tan, R.X.; Ge, H.M. *In Vitro* Reconstitution of Cinnamoyl Moiety Reveals Two Distinct Cyclases for Benzene Ring Formation. *J. Am. Chem. Soc.* **2022**, 144, 7939–7948, doi:10.1021/jacs.2c02855.
